# CSF2RB overexpression promotes the protective effects of mesenchymal stromal cells against ischemic heart injury

**DOI:** 10.7150/thno.81336

**Published:** 2023-03-13

**Authors:** Tingting Qi, Xiaoming Xu, Yongzhen Guo, Yunlong Xia, Lu Peng, Congye Li, Fengyue Ding, Chao Gao, Miaomiao Fan, Min Yu, Huishou Zhao, Yuan He, Wenli Li, Chunxu Hai, Erhe Gao, Xing Zhang, Feng Gao, Yanhong Fan, Wenjun Yan, Ling Tao

**Affiliations:** 1 Department of Cardiology, Xijing Hospital, Fourth Military Medical University, Xi'an 710032, China.; 2 School of Public Management, Northwest University, Xi'an 710127, China.; 3 Department of Toxicology, The Ministry of Education Key Lab of Hazard Assessment and Control in Special Operational Environment, Shanxi Key Lab of Free Radical Biology and Medicine, School of Public Health, The Fourth Military Medical University, Xi'an 710032, China.; 4 Department of Occupational and Environmental Health and the Ministry of Education Key Lab of Hazard Assessment and Control in Special Operational Environment, School of Public Health, Fourth Military Medical University, Xi'an 710032, China.; 5 Center for Translational Medicine, Temple University, Philadelphia, PA 19140, USA.; 6 Key Laboratory of Aerospace Medicine of the Ministry of Education, School of Aerospace Medicine, Fourth Military Medical University, Xi'an 710032, China.

**Keywords:** CSF2RB, ischemic heart injury, mesenchymal stromal cells, RNF4, STAT5

## Abstract

**Aims:** The invasive intramyocardial injection of mesenchymal stromal cells (MSCs) allows for limited repeat injections and shows poor therapeutic efficacy against ischemic heart failure. Intravenous injection is an alternative method because this route allows for repeated, noninvasive, and easy delivery. However, the lack of targeting of MSCs hinders the ability of these cells to accumulate in the ischemic area after intravenous injections. We investigated whether and how the overexpression of colony-stimulating factor 2 receptor beta subunit (CSF2RB) may regulate the cardiac homing of MSCs and their cardioprotective effects against ischemic heart failure.

**Methods and Results:** Adult mice were subjected to myocardial ischemia/reperfusion (MI/R) or sham operations. We observed significantly higher CSF2 protein expression and secretion by the ischemic heart from 1 day to 2 weeks after MI/R. Mouse adipose tissue-derived MSCs (ADSCs) were infected with adenovirus harboring CSF2RB or control adenovirus. Enhanced green fluorescent protein (EGFP)-labeled ADSCs were intravenously injected into MI/R mice every three days for a total of 7 times. Compared with ADSCs infected with control adenovirus, intravenously delivered ADSCs overexpressing CSF2RB exhibited markedly increased cardiac homing. Histological analysis revealed that CSF2RB overexpression significantly enhanced the ADSC-mediated proangiogenic, antiapoptotic, and antifibrotic effects. More importantly, ADSCs overexpressing CSF2RB significantly increased the left ventricular ejection fraction and cardiac contractility/relaxation in MI/R mice. *In vitro* experiments demonstrated that CSF2RB overexpression increases the migratory capacity and reduces the hypoxia/reoxygenation-induced apoptosis of ADSCs. We identified STAT5 phosphorylation as the key mechanism underlying the effects of CSF2RB on promoting ADSC migration and inhibiting ADSC apoptosis. RNA sequencing followed by cause-effect analysis revealed that CSF2RB overexpression increases the expression of the ubiquitin ligase RNF4. Coimmunoprecipitation and coimmunostaining experiments showed that RNF4 binds to phosphorylated STAT5. RNF4 knockdown reduced STAT5 phosphorylation as well as the antiapoptotic and promigratory actions of ADSCs overexpressing CSF2RB.

**Conclusions:** We demonstrate for the first time that CSF2RB overexpression optimizes the efficacy of intravenously delivered MSCs in the treatment of ischemic heart injury by increasing the response of the MSCs to a CSF2 gradient and CSF2RB-dependent STAT5/RNF4 activation.

## Introduction

Cardiovascular disease is the predominant cause of death worldwide. In 2019, ~18.6 million deaths around the world were attributed to cardiovascular disease, and this number represented an increase of 17.1% from 2010 1. Although technological and pharmaceutical advances have enhanced patient survival after acute myocardial infarction (MI), both the incidence and prevalence of heart failure (HF) attributable to MI have continually increased 2, 3.

Cell therapy is emerging as a promising new approach for the treatment of HF. Among the various cell types that have been investigated in the context of HF, mesenchymal stromal cells (MSCs) have been most extensively studied and hold the greatest potential for clinical application in the near future 4. Over the years, different routes of cell therapy delivery, such as intracoronary injection, systemic intravenous injection, and intramyocardial injection, have emerged and advanced the field 5. Compared to other delivery routes, intravenous injection has the prominent superiority of repeated, noninvasive, and easy operation 6, 7. Modest beneficial effects of intravenous cell therapy on cardiac function have been observed in a variety of experimental settings (acute MI, chronic MI, and nonischemic cardiomyopathy) 8. However, intravenously injected MSCs home to ischemic myocardial tissues inefficiently, limiting the effective implementation of MSC therapy 9. Therefore, novel strategies that increase the cardiac homing of intravenously delivered MSCs will improve cardiac cell therapy.

For stem cells, the word 'homing' describes the ability of stem cells to find their target organ via the bloodstream. 'Homing' directs stem cell migration via different signaling pathways, which are regulated by secreted chemokines or growth factor receptors that are expressed on the surfaces of stem cells 10. After MI, inflammation triggers the release of homing-related chemokines and cytokines, such as SDF-1, CCL2, and CCL7 11-13. Colony-stimulating factor 2 (CSF2), which is also called granulocyte-macrophage colony-stimulating factor (GM-CSF), is a multifunctional growth factor that controls leukocyte production, proliferation, differentiation, and survival 14. Although CSF2 upregulation is detrimental to post-MI cardiac remodeling 15, 16, we and others have shown that CSF2 and its receptor CSF2RB may act as a chemokine/chemokine receptor pair to enhance the cardiac homing of intravenously delivered MSCs 17, 18. However, the expression of CSF2RB in MSCs is very low, and drug-mediated CSF2RB protein upregulation is quite limited. The present study determined whether CSF2RB modification can enhance the effects of engrafted MSCs in repairing ischemic heart injury and investigated the underlying molecular mechanisms.

## Methods

### Animals

The animal experiments were approved by the Animal Care and Use Committee of the Fourth Military Medical University (Xi'an, China). Adult male C57BL/6J mice (10-12 weeks) and Sprague-Dawley pups (1-2 days old) were purchased from the Laboratory Animal Center of the Fourth Military Medical University. EGFP-expressing transgenic mice on a C57BL/6J background were purchased from The Jackson Laboratory (Stock 003291) 17. The animals were used in accordance with the regulations of the National Institutes of Health (NIH) regarding the use of laboratory animals.

### Myocardial ischemia/reperfusion (MI/R) model

To establish a mouse model of MI/R, male C57BL/6J mice were anesthetized by inhalation of 1-2% isoflurane and fixed on a thermostatic operating table. The left anterior descending coronary artery was ligated for 2 hours, followed by reperfusion 19. Sham control animals were treated by passing a suture around the left anterior descending coronary artery without ligation.

### Isolation and culture of ADSCs

ADSCs were isolated from epididymal adipocytes of adult male C57BL/6J mice as we previously described 20. The mice were anesthetized, and then, the epididymal adipose tissue was removed. After several washes with phosphate-buffered saline (PBS), the vessels were excised under a dissecting microscope. The remaining adipose tissue was minced into small pieces, added to 0.1% collagenase I that had been preprepared, digested at 37°C for 90 minutes, and centrifuged at 600 × g for 10 minutes. Subsequently, the cells were treated with red blood cell lysis buffer and cultured in α-MEM supplemented with 10% fetal bovine serum and 1% penicillin‒streptomycin. Six hours after plating the cells, the medium was replaced to remove the nonadherent cells. The medium was changed every 2 days. Cells from passages 2-3 were used in all the experiments.

### Adenovirus construction and infection

Empty plasmid adenoviruses and recombinant CSF2RB adenoviruses were constructed by Likely Biotechnology. ADSCs were infected with CSF2RB adenovirus (ADSC-CSF2RB) or empty plasmid adenovirus (ADSC-NC) for 24 hours at different multiplicities of infection (MOIs). Then, fresh medium was provided, and ADSCs were incubated for another 24 h.

### Intravenous stem cell transplantation procedures

ADSCs at passage 2-3 were intravenously injected into C57BL/6J mice 17. Four groups of animals were used: the sham-operated, MI/R + Vehicle, MI/R + ADSC-NC, and MI/R + ADSC-CSF2RB groups. ADSCs were resuspended in PBS after trypsin digestion. Three days after the MI/R operation, ADSCs were prepared in a 1 mL syringe and slowly injected with a 29 G needle at a dose of 5 × 10^5^ cells per injection into the angular vein. The process was repeated another 6 times every three days. The MI/R + Vehicle group received intravenous injections of the same volume of cell-free vector within 3 weeks after surgery.

### ADSC labeling and *ex vivo* fluorescence imaging

Cultured ADSCs were labeled with the lipophilic near infrared fluorescent dye DiR (5 μM in α-MEM -10% FBS) for 20 minutes before intravenous injection 21. To assess the distribution of DiR-labeled ADSCs, the recipient mice were sacrificed, and the organs were harvested. Fluorescence optical imaging was performed with an *in vivo* imaging system (IVIS) 1000 (Perkin Elmer, USA) in the relevant channel. The number of DiR-labeled ADSCs in the myocardium was expressed by the radiation efficiency of the fluorescence intensity.

### Evaluation of cardiac function by echocardiography and hemodynamic study

M-mode images of mice were obtained by using a Vevo 2100 echocardiography machine at 1, 21, and 42 days after MI/R injury under anesthesia by the inhalation of 2% isoflurane. Cardiac hemodynamic function was evaluated 6 weeks after MI/R utilizing a Millar tip-pressure catheter 22.

### Determination of apoptosis, angiogenesis, and fibrosis

The mice were euthanized by an overdose of pentobarbital sodium (100 mg/kg, intraperitoneal injection) 23. Hearts were harvested and embedded in paraffin, and then, heart tissues were cut into 5-μm-thick sections. Cardiomyocyte apoptosis in heart tissues was evaluated by using terminal deoxynucleotidyl transferase dUTP nick-end labeling (TUNEL) staining with the *In situ* Cell Death Detection Kit (Roche, 11684817910) according to the manufacturer's instructions. The capillary density was tested by CD31 (1 : 100, ab28364, Abcam) staining and quantified by ImageJ software. A Masson's Trichrome Stain Kit (Solarbio, G1340) was used to assess myocardial fibrosis according to the manufacturer's instructions 17.

### Small interfering RNA (siRNA) transfection

The siRNA was designed to knock down the target gene. RNF4 siRNA and scramble RNA were purchased from GenePharma (Suzhou, China). ADSCs were seeded in 6-well plates or 12-well plates. When the ADSCs reached 70 - 80% confluence, they were transfected with siRNA via Lipofectamine^TM^ RNAiMAX Transfection Reagent (#13778075, Invitrogen, USA). After 8 hours of incubation (37°C), the transfection reagent-siRNA mixture was replaced with fresh conditioned medium. Western blotting or qPCR was used to confirm the knockdown of RNF4.

### Wound healing assay

ADSCs were seeded into the 2-well culture insert of a 35 mm μ-Dish (Ibidi, 81176) and incubated for 24 hours. The culture insert can create a 500 μm cell-free gap. Then, the culture insert was removed, and the ADSCs were starved with serum-free medium. Cellular migration was visualized and recorded at the indicated time points.

### Transwell migration assay

Migration assays were carried out in 24-well two-chamber plates using polycarbonate membranes with 8-μm pores (Sigma, CLS3464). The upper chamber was precoated with Matrigel (Corning, 354248; diluted by 1/3 in serum-free medium) for 2 hours in the incubator. ADSCs (2 × 10^5^) were resuspended in 200 μL of serum-free medium and seeded in the upper chamber. After serum stimulation, conditioned medium supplemented with 10% fetal bovine serum (FBS) and 1% penicillin-streptomycin serum was added to the bottom chamber. After 36 hours of incubation at 37°C, ADSCs in the upper chamber migrated to the bottom chamber. The cells that had crossed the membrane were stained with 0.1% crystal violet (Sigma, C0775) for 10 minutes, and the numbers of migrated cells were counted by microscopy (Olympus BX51).

### RNA sequencing analysis

Differential gene expression analysis was performed using RNA sequencing at Shanghai Personal Biotechnology 20. After infection with CSF2RB or NC adenovirus for 48 hours, total RNA was extracted from ADSCs via TRIzol (Invitrogen, Carlsbad, CA, USA) according to the manufacturer's instructions.

A NanoDrop spectrophotometer was used to determine the RNA quality, integrity, and concentration (Thermo Fisher Scientific). The generation of sequencing libraries was divided into the following steps. mRNA was purified from total RNA and fragmented into small pieces. First-strand cDNA was synthesized utilizing randomized oligonucleotides and SuperScript II. Then, second-strand cDNA was synthesized. An A-tailing mix and RNA index adaptors were added for end repair.

To select the preferred 300-bp cDNA fragments, the AMPure XP system (Beckman Coulter) was used to purify the library fragments. DNA fragments with adaptor molecules ligated on both ends were selectively enriched using Illumina PCR Primer Cocktail in a 15-cycle PCR step. Products were purified (AMPure XP system) and quantified using the Agilent high sensitivity DNA assay on a Bioanalyzer 2100 system (Agilent). The sequencing library was then sequenced on a HiSeq platform (Illumina) by Shanghai Personal Biotechnology Co. Ltd., China.

### Coimmunoprecipitation

ADSCs were pretreated with CSF2RB adenovirus (ADSC-CSF2RB) or NC adenovirus (ADSC-NC) adenoviruses for 2 days. The cells were washed twice with PBS and lysed with cold 1 × lysis buffer (CST #9803) supplemented with a protease inhibitor cocktail (Thermo Fisher Scientific, 78438) and a phosphatase inhibitor cocktail (AntiProtech, APT008). The lysates were supplemented with 1 mg/mL DTBP (Thermo Fisher Scientific, 20665). The mixtures were then incubated with anti-RNF4 antibody (Proteintech #17810-1-AP) and Protein A Plus UltraLink Resin (Thermo Fisher Scientific, 53142) and rocked overnight at 4 ℃. The protein A beads were washed extensively with lysis buffer. The proteins were eluted from the beads and resolved by IgG elusion buffer (Thermo Fisher Scientific, 1856202). Samples containing a reducing agent (dithiothreitol) were heated and separated by electrophoresis. After being transferred to polyvinylidene fluoride membranes, the proteins were immunoblotted with anti-p-STAT5 (CST #4322) (1/1,000) as described above.

### Statistical analysis

All the results are presented as the mean ± SEM. GraphPad Prism 8 software (GraphPad Software, USA) was used for data analysis. One-way ANOVA followed by the Bonferroni post hoc test was performed to analyze multiple groups, and Student's t test was performed to analyze two unpaired groups. Differences with p < 0.05 were considered statistically significant.

## Results

### The expression and secretion of CSF2 by the infarcted myocardium were significantly increased after MI/R

Several studies have shown that CSF2 expression is significantly increased in the infarcted area and impairs healing after MI 14-16. CSF2 also acts as an endogenous damage signal that promotes the therapeutic effects of MSCs in an endometrial ablation animal model by enhancing the multilineage differentiation and migratory capacities of these MSCs 18. To further reveal the role of CSF2 in the MSC-based treatment of ischemic hearts, we analyzed CSF2 expression, localization, and secretion in mice and/or patients with MI/R injury.

We found that CSF2 mRNA expression was significantly higher in the heart tissues of mice subjected to MI/R at 1, 3, 7, and 14 days than in the hearts of sham mice (Figure S1A). However, the expression levels of several other chemokines/cytokines that have been shown to promote MSC homing either were downregulated (SDF-1 and HGF) or showed short-term increases (CCL2 and CCL7) (Figure S1B-E). Consistently, the protein expression of CSF2 was significantly increased in peri-infarcted heart tissues at the same time points (Figure S1F). Interestingly, the numbers of CSF2-positive cells were significantly increased, and these cells were mainly located in the infarcted and peri-infarcted areas (Figure S1G). Three days after MI/R, the plasma levels of CSF2 were higher than those in sham mice (Figure S1H). We also measured the plasma CSF2 levels in patients with stable coronary artery disease (non-MI participants) and patients who successfully underwent percutaneous coronary intervention operations (reperfusion therapy) after acute MI (MI/R patients). Three days after reperfusion therapy, the plasma CSF2 levels were significantly higher in MI/R patients than in non-MI participants (Figure S1I). Taken together, these results revealed that CSF2 protein expression and secretion by the ischemic heart were elevated from 1 day to 2 weeks after MI/R.

### CSF2RB overexpression increased the cardiac homing of intravenously delivered ADSCs

Our recent investigation showed that irisin significantly increased the cardiac homing of intravenously injected MSCs in MI/R model mice by upregulating CSF2RB, one of the CSF2 receptors 17. Hypoxic stress occurs in the myocardial microenvironment after myocardial infarction and contributes to increased apoptosis, decreased cell viability and paracrine effects of MSCs 24. The surface marker expression and differentiation potential of passage 2 ADSCs were evaluated 20, 22. We subjected ADSCs to normoxia or hypoxia/reoxygenation (H/R) and found that both the mRNA expression and protein expression of CSF2RB were markedly downregulated in the H/R group (Figures S2A-B).

Next, we aimed to determine whether the overexpression of CSF2RB promotes the cardiac homing of ADSCs administered by intravenous injection. ADSCs were infected with adenovirus carrying CSF2RB (ADSC-CSF2RB) or control adenovirus (ADSC-NC). Infection with adenovirus-CSF2RB increased CSF2RB protein expression in an MOI-dependent manner. Adenovirus-CSF2RB was delivered at MOI = 50 for further studies because it significantly increased CSF2RB protein expression in ADSCs without affecting cell viability (Figures S2C-E).

**Figure 1:**
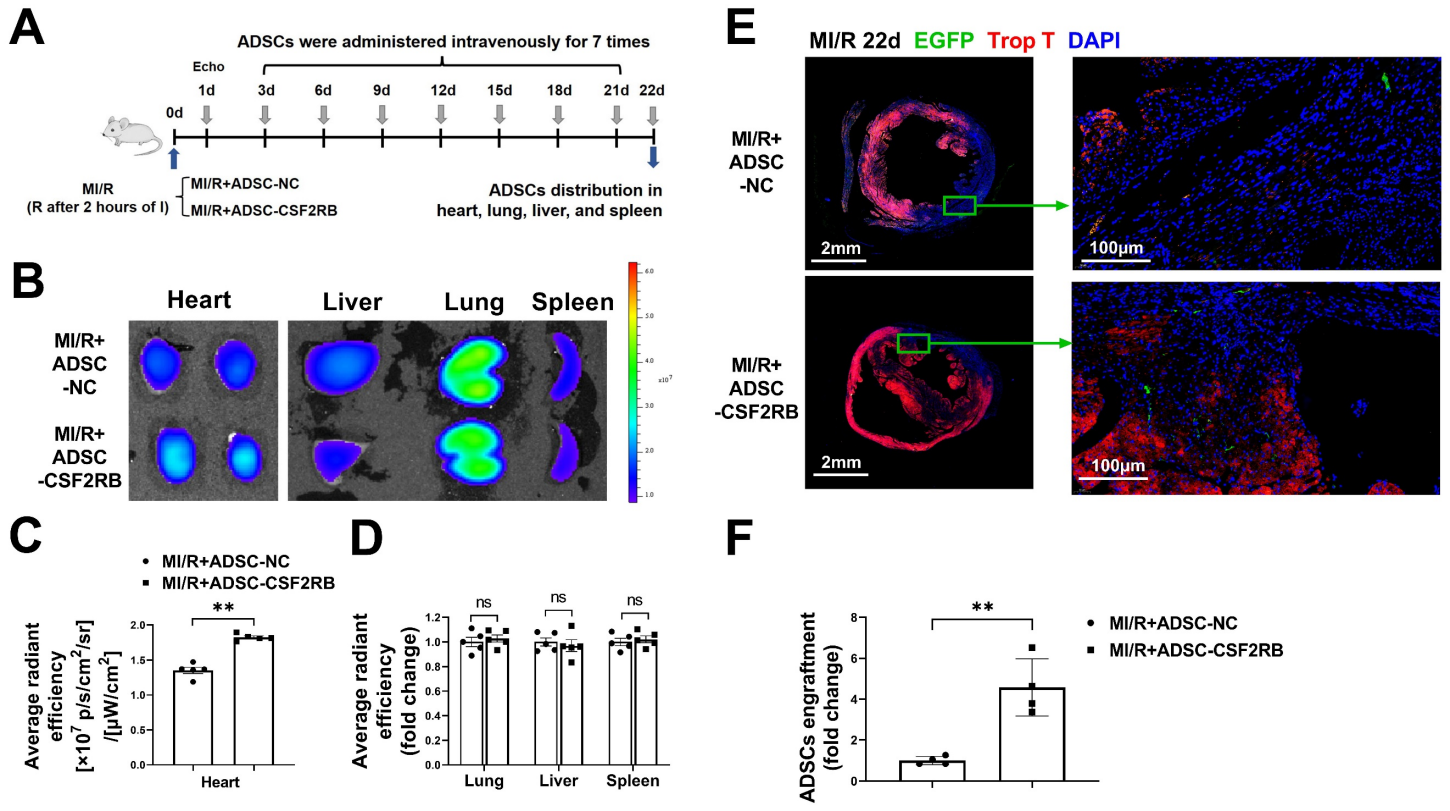
** CSF2RB overexpression increased the cardiac homing of intravenously delivered ADSCs.** (A) Flow chart of the experimental design and schedule for the cardiac homing studies using EGFP-labeled ADSC-NC and ADSC-CSF2RB. MI/R model mice were randomly assigned to receive an intravenous injection of 5 × 10^5^ ADSCs per mouse every 3 days for a total of 7 times, and the number of ADSCs in the heart, lung, liver, and spleen was counted on day 22. I, ischemia; R, reperfusion. (B-D) Representative images (B) and quantification of the fluorescence intensity of DiR-labeled ADSCs in the heart (n = 5 mice per group). (C), lung, liver, and spleen (D) on day 22 (n = 5 mice per group). The data are shown as the average radiant efficiency. (E-F) Representative images (E) and quantification (F) of EGFP-ADSCs in the peri-infarcted area of MI/R hearts on day 22. The heart sections were immunostained for troponin T (red) and DAPI (blue). The engrafted ADSCs were EGFP positive (white arrows, n = 4 mice per group). The data were analyzed by unpaired, 2-tailed Student's t test. ADSC-NC, ADSCs infected with adenovirus-control (MOI = 50) for 2 days; ADSC-CSF2RB, ADSCs infected with adenovirus-CSF2RB (MOI = 50) for 2 days. **p < 0.01. ns, not significant.

C57BL/6J mice were subjected to long-term MI/R injury, which effectively recapitulates the progression of human disease (Figure 1A). MI/R animals that had a left ventricular ejection fraction (LVEF) > 45% 1 day after the operation were excluded from the study because of the limited infarcted area 17, 25, 26. Beginning 3 days after MI/R, the mice were administered either ADSCs infected with adenovirus-CSF2RB (ADSC-CSF2RB, intravenous injection of 5 × 10^5^ cells every three days after the MI/R operation for a total of 7 times) or ADSCs infected with adenovirus-control (ADSC-NC). ADSC distribution in the heart, lung, liver, and spleen was analyzed 22 days after MI/R (Figure 1A).

We performed two independent experiments to evaluate the ADSC distribution. First, ADSCs were labeled with the lipophilic near infrared fluorescent dye DiR. Five mice from each of the MI/R + ADSC-NC and MI/R + ADSC-CSF2RB groups were sacrificed to measure the DiR signal by fluorescence imaging analysis. There was a significantly higher DiR signal in the heart tissues of MI/R + ADSC-CSF2RB mice (Figure 1B-C). Equal DiR signals were observed in the lung, liver, and spleen tissues of the two groups (Figure 1D). Second, we used ADSCs that were isolated from enhanced green fluorescent protein (EGFP) transgenic mice (ADSC-GFP) for intravenous injection. EGFP immunostaining was performed to quantify the numbers of EGFP-positive cells in the heart, lung, liver, and spleen sections. Consistently, CSF2RB overexpression significantly increased the cardiac homing of intravenously administered ADSC-GFP (Figure 1E-F). It is important to note that nearly all ADSC-GFP were found in troponin T-negative heart tissues, which were the infarcted areas (Figure 1E). However, CSF2RB overexpression did not significantly alter ADSC-GFP distribution in the lung, liver, and spleen tissues (Figure S3). Collectively, these results demonstrated that CSF2RB overexpression specifically increased the cardiac engraftment of intravenously delivered ADSCs.

### CSF2RB overexpression in ADSCs enhanced the ability of ADSCs to improve cardiac function in MI/R model mice

**Figure 2:**
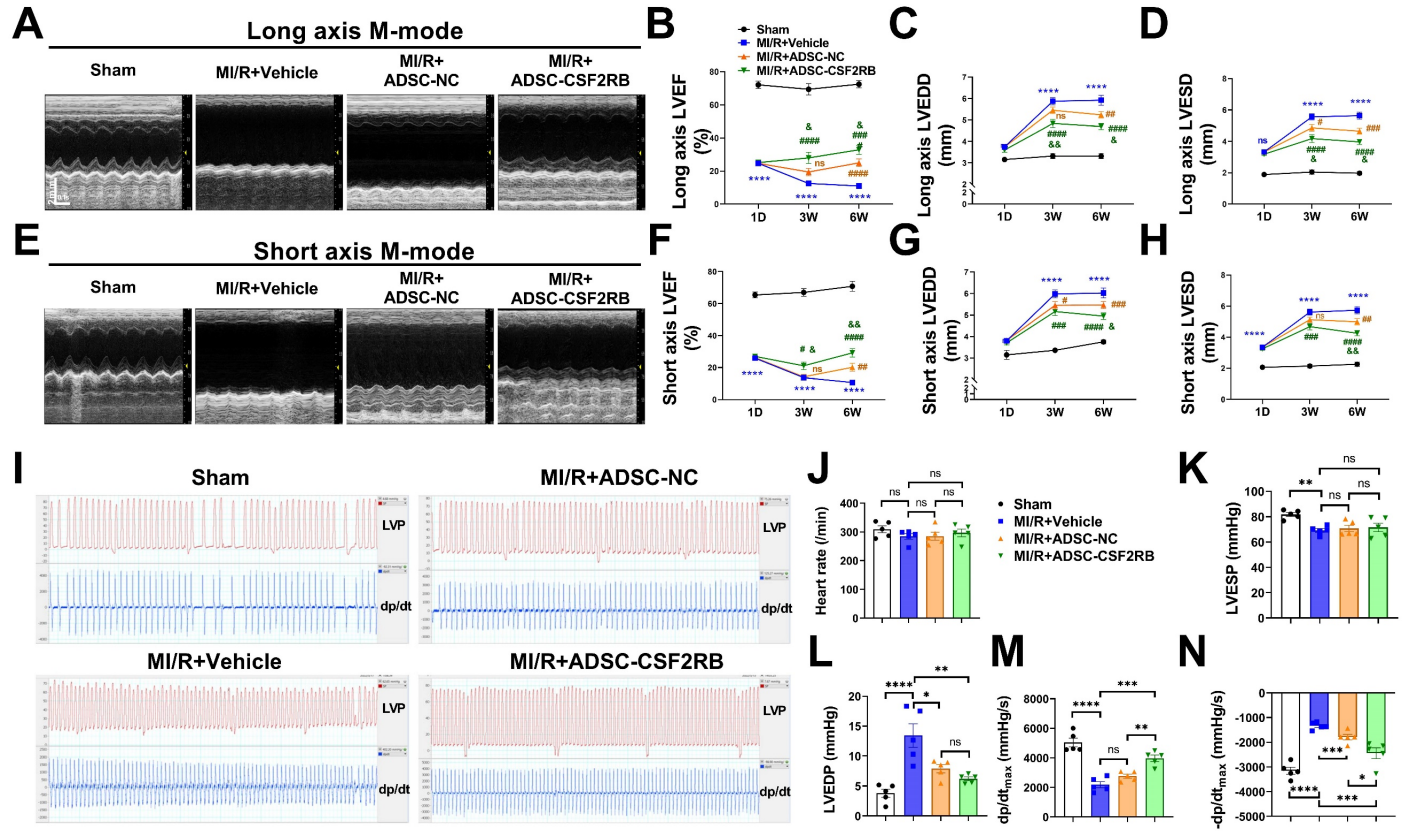
** CSF2RB overexpression enhanced the effect of intravenously delivered ADSCs in improving post-MI/R cardiac function.** (A) Representative long-axis M-mode echocardiographic images 6 weeks after MI/R. (B-D) Left ventricular ejection fraction (LVEF, B), left ventricular end-diastolic diameter (LVEDD, C), and left ventricular end-systolic diameter (LVESD, D) were evaluated by long-axis M-mode echocardiography. (E) Representative short-axis M-mode echocardiographic images 6 weeks after MI/R. (F-H) LVEF (F), LVEDD (G), and LVESD (H) were evaluated by short-axis M-mode echocardiography. n = 8, 28, 38, 28 at 1 day; n = 8, 19, 25, 21 at 3 weeks; n = 7, 19, 24, 21 at 6 weeks. ****P vs. Sham, ^#^*P,* ^##^*P,* ^###^*P,* and ^####^*P* vs. MI/R + Vehicle, ^&^*P* and ^&&^*P* vs. MI/R + ADSC-NC. (I) Representative tracings of left ventricle pressure (LVP) and dp/dt 6 weeks after MI/R. (J) Heart rate. (K) Left ventricular end-systolic pressure (LVESP). (L) Left ventricular end-diastolic pressure (LVEDP). (M) Maximal rates of LVP rise (dp/dt_max_). (N) Maximal rates of LVP decrease (-dp/dt_max_). n = 5. The data were analyzed by 1-way ANOVA, followed by Bonferroni post hoc test. ADSC-NC, ADSCs infected with adenovirus-control (MOI = 50) for 2 days; ADSC-CSF2RB, ADSCs infected with adenovirus-CSF2RB (MOI = 50) for 2 days. *p < 0.05, **p < 0.01, ***p < 0.001, ****p < 0.0001, ns, not significant.

Next, we assessed the cardioprotective effects of intravenously delivered ADSCs on mice post MI/R. ADSCs were isolated from donor C57BL/6J mice and infected with adenovirus-CSF2RB (ADSC-CSF2RB) or adenovirus-control (ADSC-NC) for 2 days. C57BL/6J mice were randomly divided into the following 4 groups: the Sham, MI/R + Vehicle, MI/R + ADSC-NC, and MI/R + ADSC-CSF2RB groups. Echocardiography was performed 1 day after MI/R. After excluding mice with an LVEF > 45% 1 day after the MI/R operation, the LVEF, left ventricular end-diastolic diameter (LVEDD), and left ventricular end-systolic diameter (LVESD) were consistent among the MI/R model mice in the different groups (Figure 2A-H). Then, the mice in the MI/R + ADSC-NC and MI/R + ADSC-CSF2RB groups received intravenous injections of either 5 × 10^5^ ADSC-NC or ADSC-CSF2RB every three days for a total of 7 times (Figure 1A).

Three weeks after MI/R, the intravenous delivery of ADSC-NC did not significantly increase the LVEF, but it slightly decreased the LVEDD and LVESD compared with those in the MI/R + Vehicle group, as determined by both long- and short-axis M-mode echocardiography (Figure 2A-H). In contrast, ADSC-CSF2RB treatment significantly increased the LVEF and decreased the LVEDD and LVESD (at 3 weeks) compared with those in the MI/R + Vehicle group (Figure 2A-H). When we re-evaluated echocardiography at 6 weeks after MI/R (3 weeks after the last cell injection), we found that both ADSC-NC and ADSC-CSF2RB significantly increased LVEF and decreased the LVEDD/LVESD. Importantly, the mice in the ADSC-CSF2RB group showed further improvement in LVEF and LVEDD/LVESD compared to those in the MI/R + ADSC-NC group.

Cardiac function was further determined by hemodynamic measurement using Millar catheterization 6 weeks after MI/R. There were no significant differences in heart rate among the mice in these 4 groups (Figure 2I-J). At 6 weeks post-MI/R, the mice that were treated with vehicle showed significant decreases in LVESP and dP/dt_max_ and increases in LVEDP and -dP/dt_max_ compared to those of the sham mice (Figure 2K-N). ADSC-NC treatment significantly decreased the LVEDP and -dP/dt_max_ but did not significantly increase the LVESP or dP/dt_max_. However, compared with the vehicle, ADSC-CSF2RB nearly fully restored the LVESP, LVEDP, and ±dP/dt_max_ (Figure 2K-N). Moreover, we found that ADSC-CSF2RB resulted in greater improvement in cardiac function than ADSC-NC (Figure 2K-N). Taken together, these results clearly showed that intravenously delivered ADSCs overexpressing CSF2RB enhanced ADSC-mediated improvements in cardiac function in a long-term MI/R mouse model.

### CSF2RB overexpression in ADSCs enhanced the anti-remodeling effects of the cells in mouse hearts after MI/R

**Figure 3:**
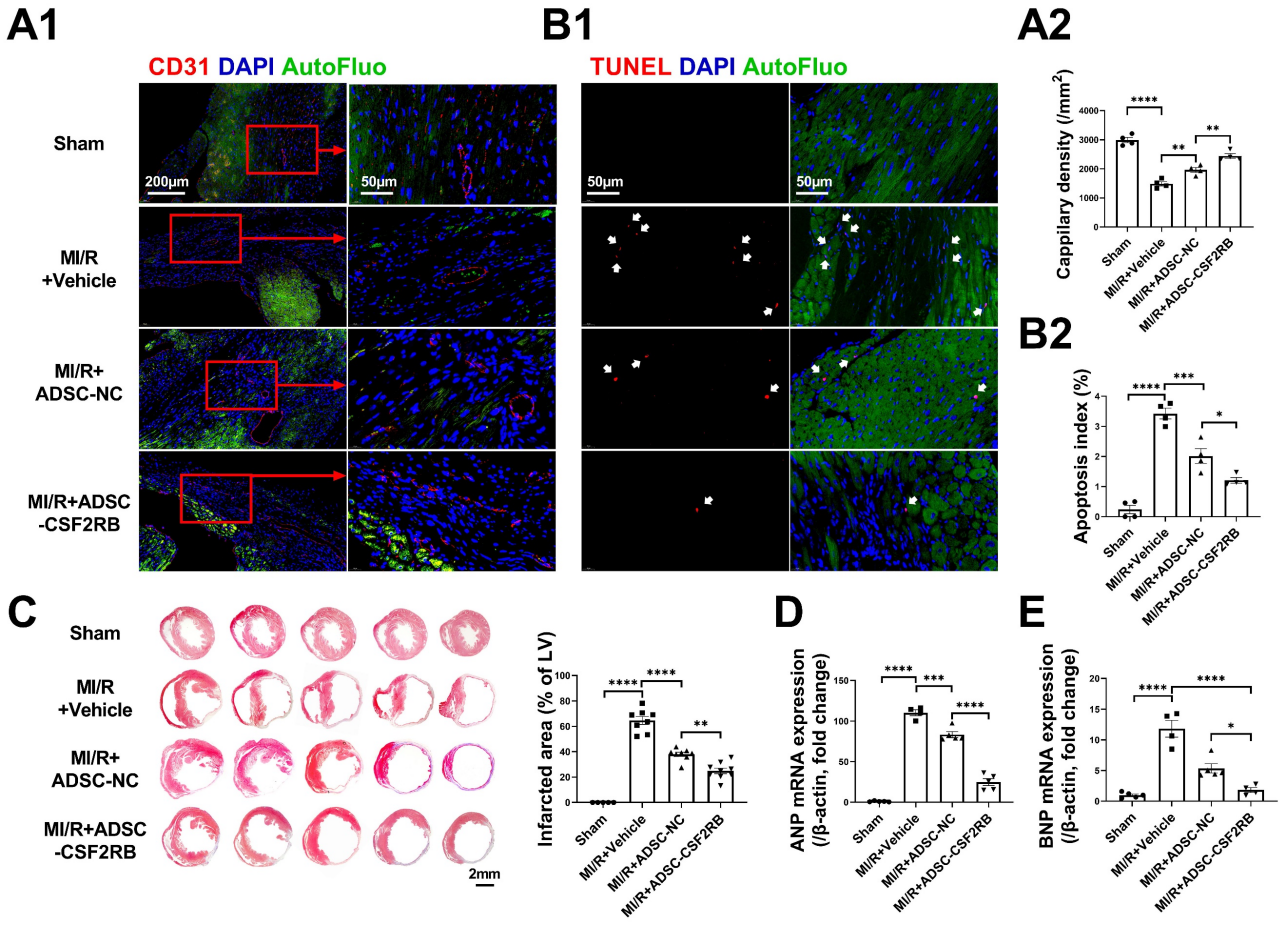
** Intravenous injection of ADSCs overexpressing CSF2RB (ADSC-CSF2RB) preserved capillary density and reduced cardiomyocyte apoptosis after MI/R.** (A) Representative images (A1) and quantification (A2) of capillary density in the peri-infarcted area 3 weeks after MI/R, as determined by CD31 immunofluorescence staining (n = 4 mice per group). (B) Representative images (B1) and quantification (B2) of TUNEL-positive cardiomyocytes (red, indicated by the white arrows) in the peri-infarcted area 3 weeks after MI/R (n = 4 mice per group). (C) Five sections of representative images of Masson trichrome staining and quantification of the fibrotic area of heart tissue 6 weeks after MI/R (n = 5-8 mice per group). (D-E) Quantitative polymerase chain reaction (qPCR) analysis of the mRNA expression of atrial natriuretic peptide (ANP, D) and brain natriuretic peptide (BNP, E) in heart tissues (n = 4-5 mice per group). The data in (A2) were analyzed by 1-way ANOVA, followed by Bonferroni post hoc test. The other data were analyzed by the Kruskal-Wallis test followed by Dunn's post hoc test. ADSC-NC, ADSCs infected with adenovirus-control (MOI = 50) for 2 days; ADSC-CSF2RB, ADSCs infected with adenovirus-CSF2RB (MOI = 50) for 2 days. *p < 0.05, **p < 0.01, ***p < 0.001, ****p < 0.0001, ns, not significant.

The cardioprotective effects of MSCs have been found to be associated with enhanced cardiac angiogenesis and reduced cardiomyocyte apoptosis 27, 28. Three weeks after MI/R, the capillary density in the infarcted heart areas was markedly decreased compared with that in sham hearts, as determined by CD31 immunofluorescence staining (Figure 3A). After 7 ADSC-NC administrations, the capillary density was significantly increased in the ADSC-NC group and was further increased in the ADSC-CSF2RB group. TdT-mediated dUTP nick-end labeling (TUNEL) staining was performed to assess apoptosis in the peri-infarct regions of the hearts. ADSC-NC treatment significantly decreased the number of TUNEL + cardiomyocytes compared to vehicle treatment, while ADSC-CSF2RB treatment further reduced the apoptotic index (Figure 3B). Masson's trichrome staining of the transverse plane 6 weeks after MI/R revealed that ADSC-CSF2RB were more efficient in reducing the myocardial fibrotic area than ADSC-NC (Figure 3C). Moreover, cardiac remodeling was assessed at the molecular level. Quantitative polymerase chain reaction (qPCR) showed that the mRNA levels of atrial natriuretic peptide (ANP) and brain natriuretic peptide (BNP) were markedly downregulated by ADSC-NC treatment. Compared to ADSC-NC, ADSC-CSF2RB further decreased ANP and BNP mRNA expression (Figure 3D-E). Collectively, these data demonstrated that intravenously delivered CSF2RB-overexpressing ADSCs enhanced ADSC-mediated anti-remodeling effects.

### CSF2RB overexpression increased the migration and antiapoptotic effects of ADSCs without affecting the paracrine function of ADSCs

**Figure 4:**
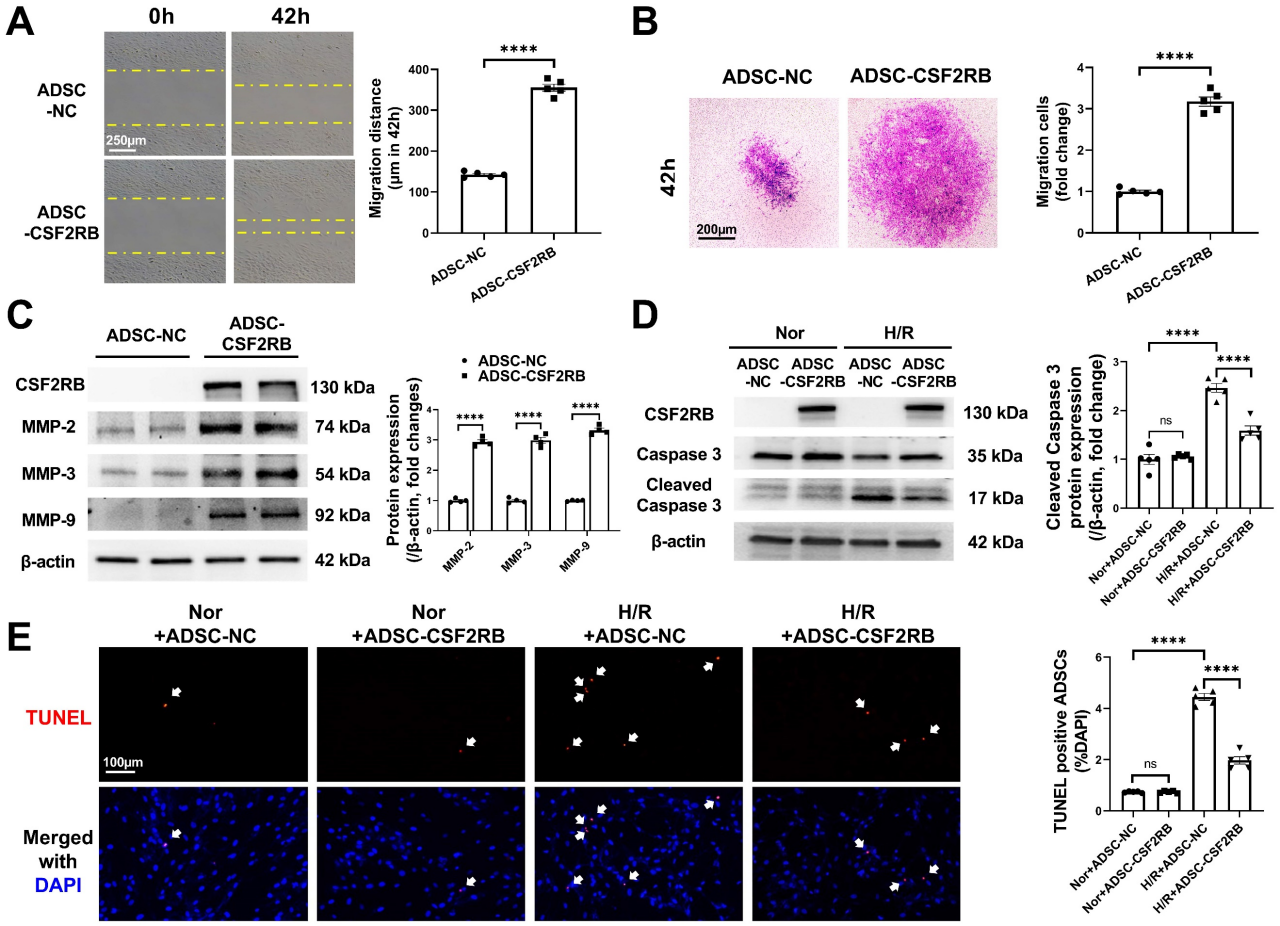
** CSF2RB overexpression increased the migration and antiapoptotic effect of ADSCs.** (A) ADSC migratory capacity was analyzed by wound healing assay for 42 hours. (B) ADSC migratory capacity was analyzed by Matrigel-coated Transwell assay for 42 hours. ADSCs that migrated through the Matrigel were stained with crystal violet. In (A) and (B), ADSCs were infected with adenovirus-CSF2RB (ADSC-CSF2RB) or adenovirus-control (ADSC-NC) for 48 hours. n = 5. (C) Western blotting and quantification of MMP-2, MMP-3, and MMP-9 protein expression in ADSCs. n = 4. (D) Protein expression of cleaved caspase-3 in ADSCs. n = 5. (E) Representative images of TUNEL-positive nuclei (red) in ADSCs. DAPI was used to stain the cell nuclei (blue). n = 5. In (D) and (E), ADSC-NC and ADSC-CSF2RB were placed under normoxic (Nor) conditions or subjected to hypoxia for 13 hours followed by reoxygenation for another 1 hour (H/R). The data in (A-C) were analyzed by unpaired, 2-tailed Student's t test. The other data were analyzed by 1-way ANOVA, followed by Bonferroni post hoc test. ****p < 0.0001; ns, not significant.

We next sought to determine the cellular mechanism(s) underlying the increased cardiac homing and reparative effects of ADSC-CSF2RB. Because the homing of stem cells is largely dependent on their migratory capacity 17, 18, 20, 28, 29, we first compared the migration of ADSC-NC and ADSC-CSF2RB. Wound healing and Matrigel-coated Transwell experiments demonstrated that ADSC-CSF2RB exhibited a markedly better migratory capacity than ADSC-NC (Figure 4A-B). Matrix metallopeptidases (MMPs) have been previously reported to play an important role in the process of MSC migration 28, 30. We found that CSF2RB overexpression significantly increased the protein expression of MMP-2, MMP-3, and MMP-9 in ADSCs (Figure 4C).

After homing to the infarcted heart, stem cells encounter a harsh ischemic microenvironment that affects cell retention. Therefore, we evaluated the anti-apoptotic potential of ADSC-NC and ADSC-CSF2RB. Both ADSC-NC and ADSC-CSF2RB were subjected to normoxia or H/R. Immunoblotting demonstrated that after exposure to H/R, the protein expression of cleaved caspase-3 was significantly reduced in ADSC-CSF2RB compared to ADSC-NC (Figure 4D). The numbers of TUNEL+ nuclei in ADSC-CSF2RB were significantly lower than those of ADSC-NC after H/R (Figure 4E).

It is now widely accepted that transplanted MSCs improve the function of injured hearts via paracrine mechanisms 4. We next determined whether CSF2RB could improve the paracrine functions of ADSCs. The effects of fresh medium (FM), conditioned medium (CM) from ADSC-NC (ADSC-NC-CM), and CM from ADSC-CSF2RB (ADSC-CSF2RB-CM) on the apoptosis of H/R-exposed neonatal rat ventricular cardiomyocytes (NRVCs) were determined. Compared with FM, ADSC-NC-CM significantly decreased NRVC apoptosis. However, ADSC-CSF2RB-CM did not further reduce NRVC apoptosis compared with ADSC-NC-CM (Figure S4A). Similarly, ADSC-NC-CM and ADSC-CSF2RB-CM equally increased the tube formation ability of rat coronary artery endothelial cells (Figure S4B). The number of extracellular vesicles (EVs) had no significant difference between ADSC-NC-CM and ADSC-CSF2RB-CM (Figure S4C). Thus, these data demonstrated that CSF2RB overexpression increases the migration and anti-apoptosis of ADSCs without affecting their paracrine function.

### CSF2RB overexpression increased the migration and antiapoptotic effects of ADSCs via STAT5 phosphorylation

**Figure 5:**
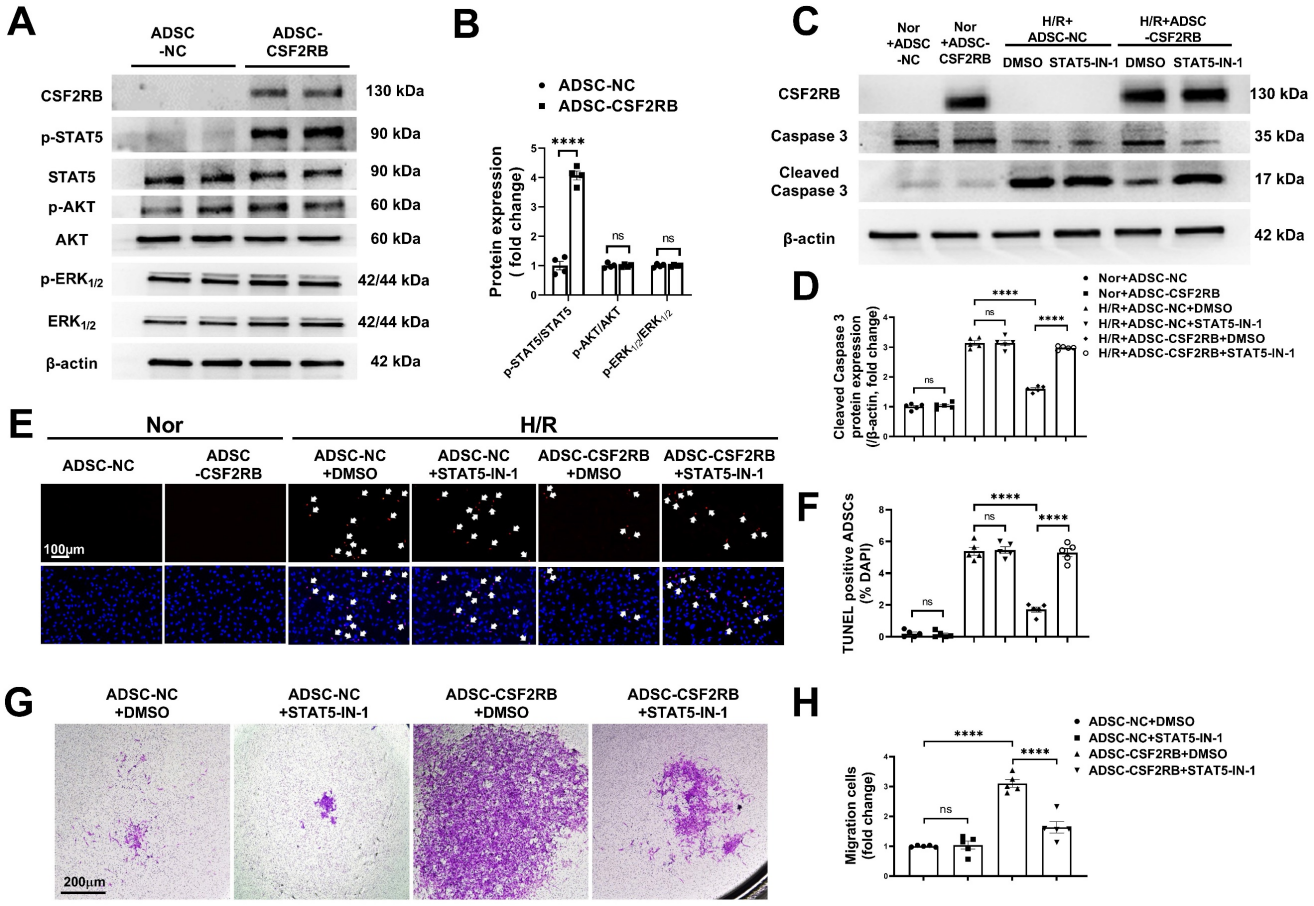
** CSF2RB overexpression reduced ADSC apoptosis and improved ADSC migration via the phosphorylation of STAT5.** (A-B) Western blotting (A) and quantification of the protein expression of p-STAT5, p-AKT, and p-ERK1/2 (B) in ADSCs. ADSCs were infected with adenovirus-CSF2RB (ADSC-CSF2RB) or adenovirus-control (ADSC-NC) for 48 hours. n = 4. (C-D) Protein expression of cleaved caspase-3 in ADSCs. n = 5. (E) Representative images of TUNEL-positive nuclei (red) in ADSCs. DAPI was used to stain the cell nuclei (blue). (F) Quantification of the number of TUNEL-positive ADSCs. n = 5. In (C) through (F), ADSC-NC and ADSC-CSF2RB were treated with DMSO or STAT5-IN-1 (100 µM) for 24 hours and were then placed under normoxic (Nor) conditions or subjected to hypoxia for 13 hours followed by reoxygenation for another 1 hour (H/R). (G-H) ADSC migratory capacity was analyzed by Matrigel-coated Transwell assay. ADSC-NC and ADSC-CSF2RB were treated with DMSO or STAT5-IN-1 (100 µM) for 24 hours. ADSCs that migrated through the Matrigel were stained with crystal violet. n = 5. The data in (B) were analyzed by unpaired, 2-tailed Student's t test. The other data were analyzed by 1-way ANOVA, followed by Bonferroni post hoc test. ****p < 0.0001, ns, not significant.

CSF2 signaling activates several intracellular signaling pathways, including the phosphatidylinositol 3-kinase/protein kinase B (PI3K/AKT), extracellular signal-regulated kinase 1/2 (ERK1/2), and Janus kinase 2 (JAK2)/signal transducer and activator of transcription 5 (STAT5) pathways 16, 18, 31. We quantified AKT, ERK1/2, and STAT5 phosphorylation in ADSC-NC and ADSC-CSF2RB. Here, we showed that CSF2RB overexpression significantly increased STAT5 phosphorylation without affecting AKT or ERK1/2 phosphorylation (Figure 5A-B).

To determine whether the migration and antiapoptotic effects of cells overexpressing CSF2RB are regulated by STAT5 signaling, ADSC-CSF2RB were treated with STAT5-IN-1 (a STAT5 inhibitor). CSF2RB overexpression failed to increase STAT5 phosphorylation in the presence of STAT5-IN-1 for 24 and 48 hours (Figure S5A and B). Importantly, in the presence of STAT5-IN-1, ADSC-CSF2RB showed significantly increased cleaved caspase-3 protein expression after H/R injury (Figure 5C-D). After H/R injury, there were more TUNEL+ nuclei in the ADSC-CSF2RB + STAT5-IN-1 group than in the ADSC-CSF2RB + DMSO group (Figure 5E-F). A Matrigel-coated Transwell assay demonstrated that STAT5-IN-1 attenuated CSF2RB-enhanced migratory capacity (Figure 5G-H). STAT5-IN-1 also significantly reduced MMP-2, MMP-3, and MMP-9 protein expression in ADSC-CSF2RB (Figure S5C and D). Thus, these data demonstrated that STAT5 activation mediates the effects of CSF2RB overexpression on the migration and survival of ADSCs.

### CSF2RB-STAT5-dependent RNF4 upregulation was responsible for CSF2RB-mediated increases in ADSC survival and migration

**Figure 6:**
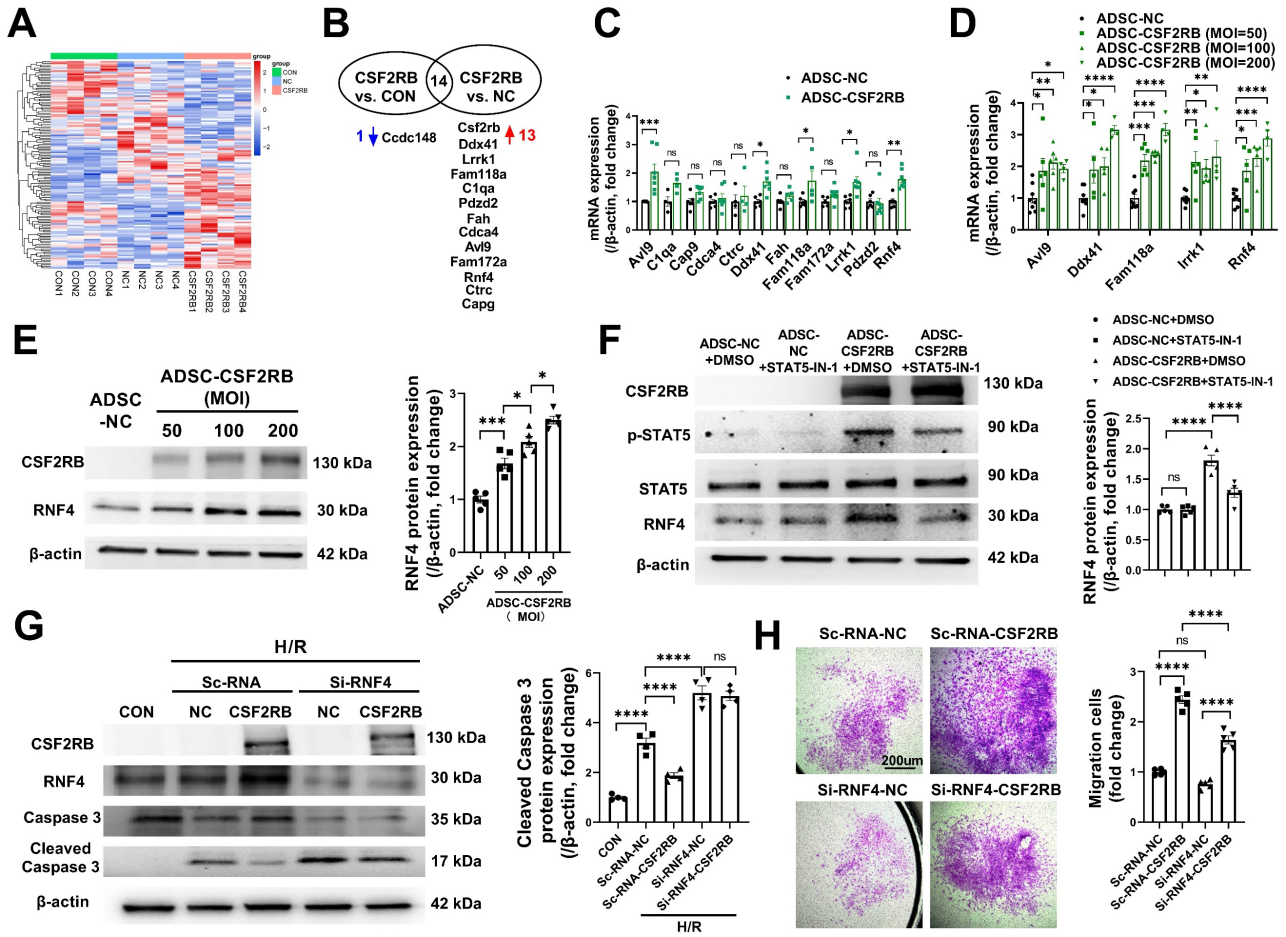
** CSF2RB overexpression reduced ADSC apoptosis and improved ADSC migration via STAT5-dependent RNF4 upregulation.** (A) Cluster analysis of the differentially expressed genes identified by RNAseq of ADSCs treated with blank control (CON), adenovirus-control (ADSC-NC), or adenovirus-CSF2RB (ADSC-CSF2RB) for 48 hours. (B) The 13 upregulated genes and 1 downregulated gene in ADSC-CSF2RB cells compared to ADSC-NC cells. (C) Real-time PCR analysis of the mRNA expression of the 12 upregulated genes in ADSC-CSF2RB (n = 4-7 mice per group). (D) Real-time PCR analysis of the mRNA expression of Avl9, Ddx41, Fam118a, Lrrk1, and RNF4 (n = 4-8 mice per group). (E) Western blotting and quantification of RNF4 protein expression in ADSC-NC and ADSC-CSF2RB that were infected at different MOIs. n = 5. (F) Protein expression of RNF4 in ADSC-NC and ADSC-CSF2RB treated with DMSO or STAT5-IN-1. n = 5. (G) Protein expression of cleaved caspase-3 in ADSCs. n = 5. (H) ADSC migratory capacity was evaluated by Matrigel-coated Transwell assay. ADSCs that migrated through the Matrigel were stained with crystal violet. n = 5. The data in (C) and (D) were analyzed by unpaired, 2-tailed Student's t test. The other data were analyzed by 1-way ANOVA, followed by Bonferroni post hoc test. *p < 0.05, **p < 0.01, ***p < 0.001, ****p < 0.0001, ns, not significant.

We then used a nonbiased approach via RNA sequencing of RNA samples from blank control ADSCs (ADSC-CON), ADSC-NC, and ADSC-CSF2RB (Figure 6A). Genes whose expression had a p value ≥ 0.05 between ADSC-CSF2RB and ADSC-NC or between ADSC-CSF2RB and ADSC-CON were excluded. Genes with a basal read count ≤ 50 were also excluded. We then calculated the fold change in gene expression between ADSC-CSF2RB and ADSC-NC and between ADSC-CSF2RB and ADSC-CON, and we filtered genes with a fold change > 1.5 or < 0.67. We found that 13 genes were upregulated and 1 gene was downregulated in ADSC-CSF2RB cells compared to ADSC-NC cells (Figure 6B, Table S1). Using qPCR analysis, 5 genes were confirmed to be upregulated in ADSC-CSF2RB cells (Figure 6C, Table S2). Among these 5 genes, only RING finger protein 4 (RNF4) transcription was increased by CSF2RB overexpression in a dose-dependent manner (Figure 6D), suggesting specific regulation. We did not focus on the other 4 genes because CSF2RB overexpression was not able to dose-dependently upregulate their expression (Figure 6D). We then performed immunoblotting and found that RNF4 protein expression was also increased by CSF2RB overexpression in a dose-dependent manner (Figure 6E). Moreover, STAT5-IN-1 significantly attenuated RNF4 upregulation in ADSC-CSF2RB (Figure 6F).

RNF4 belongs to a small group of RING finger E3 ubiquitin ligases that are collectively coined small ubiquitin-related modifier (SUMO)-targeted ubiquitin ligases 32. To determine the involvement of RNF4 in CSF2RB-mediated antiapoptotic and promigratory effects, ADSCs were transfected with scRNA (Sc-RNA) or short-interfering RNA (siRNA) against RNF4 (Si-RNF4) and were then infected with adenovirus-control or adenovirus-CSF2RB. Importantly, RNF4 knockdown not only increased H/R-induced ADSC apoptosis but also attenuated the protective effect of CSF2RB overexpression against ADSC apoptosis, as shown by the measurement of cleaved caspase-3 protein expression (Figure 6G). RNF4 knockdown also partially reduced the migratory capacity of ADSC-CSF2RB (Figure 6H). Collectively, these data suggest that CSF2RB upregulates RNF4 expression via STAT5 activation, protecting ADSCs from H/R-induced cell death and enhancing their migratory capacity.

### RNF4 bound to and maintained phosphorylated STAT5

**Figure 7:**
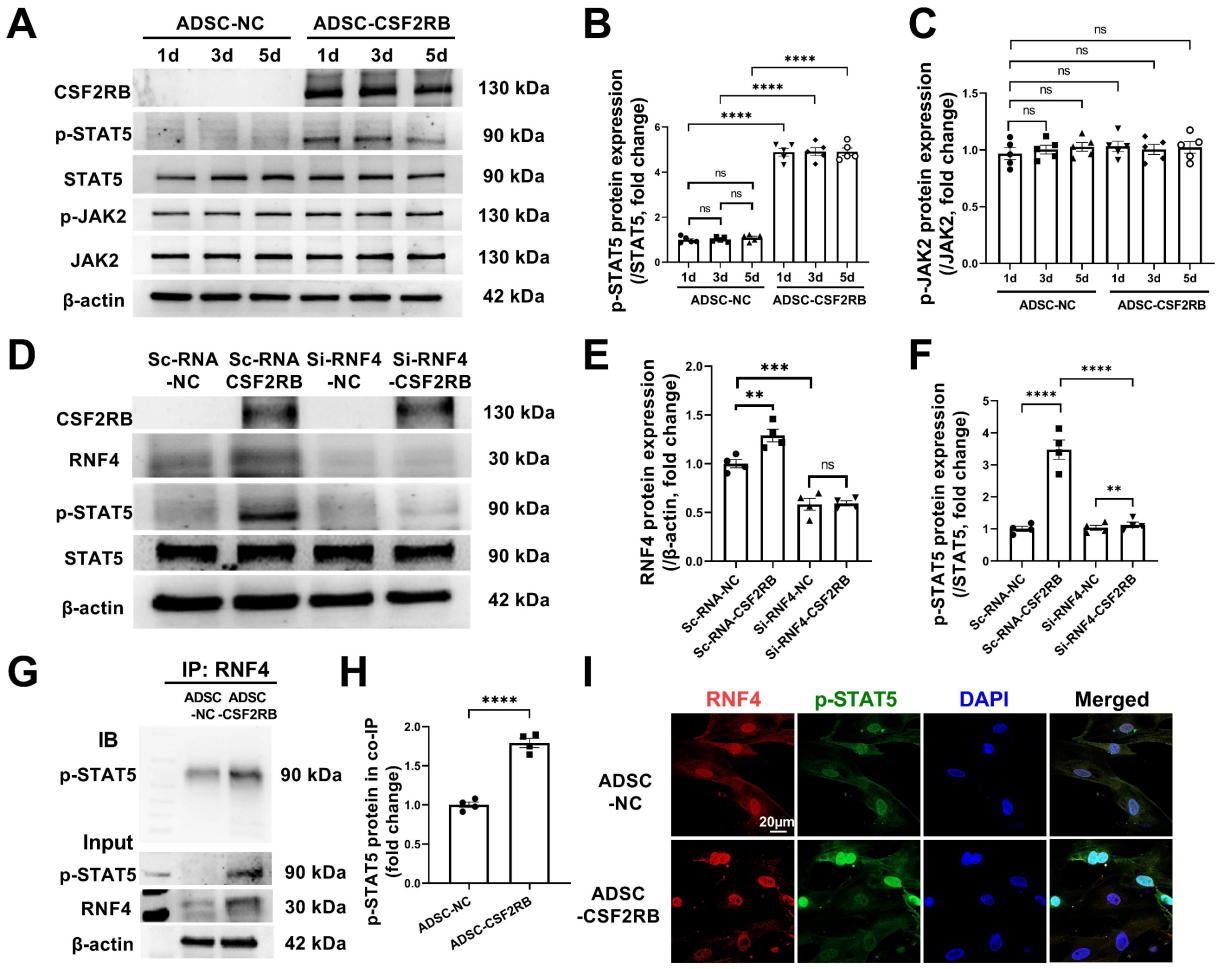
** RNF4 bound to and maintained phosphorylated STAT5.** (A-C) Western blotting (A) and quantification of p-STAT5 (B) and p-JAK2 (C) protein expression in ADSCs infected with adenovirus-control or adenovirus-CSF2RB for 1, 3, or 5 days. n = 5. (D-F) Protein expression of RNF4 and p-STAT5 in ADSCs. n = 4. ScRNA: scramble RNA. Si-RNF4: RNF4 siRNA. NC: control adenovirus. CSF2RB: CSF2RB adenovirus. (G-H) Coimmunoprecipitation (IP) and immunoblotting (IB) analysis of RNF4 with p-STAT5 in ADSC whole-cell lysates. (I) Coimmunostaining of RNF4 and p-STAT5 in ADSC-NC and ADSC-CSF2RB. n = 4. The data in (H) were analyzed by unpaired, 2-tailed Student's t test. The other data were analyzed by 1-way ANOVA, followed by Bonferroni post hoc test. **p < 0.01, ***p < 0.001, ****p < 0.0001, ns, not significant.

As our results indicated that there is a high level of phosphorylated STAT5 in ADSCs that have overexpressed CSF2RB for at least 5 days (Figure 7A-B), we aimed to determine how CSF2RB overexpression increases and maintains STAT5 phosphorylation. CSF2-dependent STAT5 phosphorylation requires the phosphorylation of JAK2 33. We thus measured the protein expression of phosphorylated and total JAK2 in ADSC-NC and ADSC-CSF2RB. Unexpectedly, CSF2RB overexpression did not alter JAK2 expression or phosphorylation in ADSCs (Figure 7A and C).

A previous study reported that the ubiquitin ligase MDM2 binds to and enhances STAT5 stability in T cells 34. It has also reported that RNF4 controls several short-lived phosphorylated proteins, including p-c-Myc, p-β-catenin, p-c-Jun, and p-eIF2α 35, 36. We found that knockdown of RNF4, a ubiquitin ligase, significantly reduced STAT5 phosphorylation in ADSC-CSF2RB without affecting STAT5 protein expression (Figure 7D-F). However, whether and how RNF4 affects STAT5 phosphorylation has never been reported. We then performed coimmunoprecipitation using ADSC-NC and ADSC-CSF2RB and found that RNF4 bound to phosphorylated STAT5, and ADSC-CSF2RB showed a significant increase in RNF4/phosphorylated STAT5 binding (Figure 7G and H). Consistently, RNF4 and phosphorylated STAT5 were found to colocalize in the nuclei of ADSC-CSF2RB according to confocal microscopy (Figure 7I). Taken together, these data demonstrated that CSF2RB overexpression increases the protein expression of RNF4, which binds to and maintains STAT5 phosphorylation.

## Discussion

The cardioprotective potential of intravascular administration of MSCs has been widely recognized in clinical trials 37-39. However, the efficacy of this treatment is limited due to the low homing and retention of these cells. It is now well accepted that therapeutic MSCs need to be recruited/retained at the site of injury in significant numbers and for a sufficient duration to exert an effect 40. Although several chemokine/chemokine receptor pairs, such as SDF-1/CXCR4, CCL2/CCR2, CCL7/CCR1, and HGF/c-Met, have been reported to enhance the homing of intravenously administered MSCs, their efficacy is quite limited 11-13, 41. There is still a lack of approaches to drive MSC homing to infarcted heart tissue.

There were four novel and notable observations in the current study (Figure 7J). First, we demonstrated that CSF2RB overexpression promotes the cardiac homing and cardioprotective effects of ADSCs that are administered via multiple intravenous injections. CSF2RB is the common signaling subunit of the cytokine receptors for IL-3, IL-5, and CSF2. We recently reported that CSF2RB upregulation by irisin enhances the cardiac homing of intravenously administered MSCs toward CSF2, identifying CSF2/CSF2RB as a chemotactic signaling axis for MSCs 17. However, MSCs lack CSF2RB expression and function. In the present study, we overexpressed CSF2RB in ADSCs and then performed multiple intravenous injections of ADSCs in a long-term MI/R mouse model. We demonstrated that CSF2RB overexpression increased the cardiac homing of intravenously injected ADSCs in post-MI/R model mice by tracking ADSCs stained with DiR or genetically labeled with GFP. CSF2RB overexpression specifically increased ADSC accumulation in the ischemic heart but not in the lung, liver, or spleen. Although the cardiac homing of intravenously delivered ADSC-NC was limited, MI/R model mice that received 7 intravenous injections of 5 × 10^5^ ADSC-NC every 3 days exhibited improved cardiac function and remodeling, as shown by increased angiogenesis as well as reduced fibrosis and cardiomyocyte apoptosis. Importantly, CSF2RB overexpression further enhanced the cardioprotective effect of ADSCs against ischemic heart injury. These results indicate that CSF2RB is an important molecule for promoting ADSC cardiac homing and reparative potential.

Second, we revealed novel cellular mechanisms that directly underlie the effects of CSF2RB overexpression on ADSCs. CSF2RB promotes cell survival, proliferation and differentiation 42, 43. In the present study, we demonstrated that ADSCs overexpressing CSF2RB exhibited increased protein expression of several MMPs and enhanced migratory properties, which facilitated ADSC migration into the infarcted heart. After homing to the ischemic heart, the cells are subjected to a harsh ischemic, hypoxic, and inflammatory microenvironment. The ability of transplanted MSCs to survive largely determines their cardioprotective effects. We found that ADSCs overexpressing CSF2RB were largely resistant to H/R-induced cell death. Therefore, CSF2RB overexpression may enhance the cardioprotective effects of ADSCs by increasing their migration and survival, which facilitate their homing and retention in ischemic heart tissue. There are two potential advantages of CSF2/CSF2RB over the abovementioned chemokine/chemokine receptors. On the one hand, the chemokine CSF2 shows a long-term increase after MI/R, forming a stable CSF2 gradient between the ischemic heart and blood stream. On the other hand, we demonstrated that CSF2RB overexpression not only increases the migratory capacity of MSCs but also reduces H/R-induced MSC apoptosis. Importantly, the CSF2RB-mediated anti-apoptotic effect in MSCs is CSF2-independent, which makes CSF2RB an accessible therapeutic target.

Third, we revealed that STAT5 phosphorylation is responsible for the effects of CSF2RB on ADSC migration and survival. STAT5 is a transcription factor that is activated by various cytokines, hormones, and growth factors. CSF2 activates STAT5 in myeloid cells 16, 31. Activated STAT5 translocates to the nucleus and regulates the transcription of target genes, affecting several biological processes 44. In MSCs, STAT5 has been shown to promote cell migration and inhibit adipocyte differentiation 29. We found that CSF2RB overexpression increases STAT5 phosphorylation in ADSCs. Administration of STAT5-IN-1 almost completely attenuated the CSF2RB-mediated upregulation of MMPs and the promigratory and antiapoptotic effects of ADSCs. Consistently, Riccioni et al. and Charlet et al. previously correlated the high expression of CSF2RB in acute myeloid leukemia patients with higher STAT5 phosphorylation 43, 45. This evidence supports the conclusion that CSF2RB overexpression enhances the promigratory and antiapoptotic effects of ADSCs in a STAT5-dependent manner.

Finally, we identified the ubiquitin ligase RNF4 as a positive regulator of STAT5 phosphorylation in ADSCs. Once activated by phosphorylation, JAK2 activates a number of downstream molecules, including STAT3 and STAT5. The JAK2-STAT5 pathway plays a particular role in cell survival and proliferation 46. Li et al. observed that phosphorylated STAT5 levels were increased in macrophages following JAK2 phosphorylation after 2-3 days of CSF2 exposure, and activated JAK2/STAT5 induced M0/M1-to-M2 transformation 33. Here, we found that CSF2RB overexpression did not alter JAK2 expression or phosphorylation in ADSCs. However, CSF2RB overexpression increased STAT5 phosphorylation for at least 5 days in ADSCs, which is largely different from CSF2 exposure. RNA sequencing analysis followed by qPCR/Western blotting demonstrated that RNF4 was significantly upregulated in ADSCs overexpressing CSF2RB. Interestingly, STAT5 inhibition reduced RNF4 protein expression, while RNF4 knockdown blocked STAT5 phosphorylation in ADSCs overexpressing CSF2RB. The loss-of-function study showed that RNF4 protects ADSCs against H/R-induced apoptosis. RNF4 directly bound to phosphorylated STAT5 in ADSC-CSF2RB cells, as determined by coimmunoprecipitation and coimmunostaining experiments. A previous study reported that RNF4 reduced cardiac fibrosis in a transverse aortic constriction mouse model 47. Our results not only provide further evidence that RNF4 is able to translate transient phosphorylation signals into long-term protein stabilization 35, 36 but also identify RNF4 as a potential target for improving MSC-based treatments to repair cardiac damage.

In summary, we identified CSF2RB overexpression as a novel method to improve the cardiac homing and survival of ADSCs in a mouse MI/R model. We delineated novel cellular and molecular mechanisms by which CSF2RB augments the cardioprotective potential of ADSCs. Multiple intravenous injections of MSCs with CSF2RB overexpression could be a novel strategy for cardiac repair after myocardial ischemic injury.

## Supplementary Material

Supplementary methods, figures and tables.
