# Supplementary material for: CSF2RB overexpression promotes the protective effects of mesenchymal stromal cells against ischemic heart injury
Source: Theranostics. 2023 Mar 13;13(6):1759–73. doi: 10.7150/thno.81336 (PMC10091875; doi:10.7150/thno.81336)
Supplement: Supplementary file 1 — Supplementary methods, figures and tables. [file thnov13p1759s1.pdf]

ONLINE SUPPLEMENTATION

Extended Methods

Study population

The patients were from the 'TARGET STEMI OCT China Trial' performed by our group (Unique identifier: NCT04150016); please visit the following website for details: https://www.clinicaltrials.gov/ct2/show/NCT04150016.

Acute myocardial infarction (AMI) patients were admitted to Xijing Hospital (Xi'an, China). We included adult patients (aged 18–75 years) who presented with typical chest pain of < 12 hours duration and ST-segment elevation of ≥ 0.1 mV in at least two or more contiguous electrocardiographic leads [1, 2]. Patients were excluded for the following reasons: cardiogenic shock at admission; active infections; systemic inflammatory disease; known hepatic, hematological, or malignant disease; end-stage renal disease (glomerular filtration rate < 15 ml/min/1.73 m^2); surgery in the previous 3 months; and lack of emergency percutaneous coronary intervention (PCI). All primary PCIs were performed by experienced experts who performed > 500 PCIs/year. After primary PCI, patients were medically treated per contemporary guidelines [3]. Following signed informed consent, blood samples were collected 3 days after the primary PCI procedure. Plasma was stored at –80°C until being assayed for blinded determination of CSF2 (Immunoway, KE1019). Patients with stable coronary artery disease were recruited as controls (non-MI participants). The study adhered to the international rules for scientific studies and the Helsinki principles. Local ethics committee approval was obtained. All subjects provided informed consent.

Evaluation of cardiac function by echocardiography

M-mode images of mice were obtained by using a Vevo 2100 echocardiography

machine at 1, 21, and 42 days after MI/R injury under anesthesia by the inhalation of 2% isoflurane. The mice were fixed on the operating table in the supine position, and an ultrasonic probe was adjusted to obtain two-dimensional images of the short axis and long axis of the left ventricle. The left ventricular end-systolic dimension (LVESD), the left ventricular end-diastolic dimension (LVEDD), the left ventricular end-systolic volume (LVESV) and the left ventricular end-diastolic volume (LVEDV) were measured. The left ventricular ejection fraction (LVEF) was automatically calculated by Vevo LAB 3.1.1 software: LVEF (%) = $100 \times [(LVEDD^3 - LVESD^3) / LVEDD^3]$. Three consecutive cardiac cycle parameters were measured in M mode and averaged.

Hemodynamic study

Cardiac hemodynamic function was evaluated 6 weeks after MI/R utilizing a Millar tip-pressure catheter [4]. The mice were anesthetized with 2-3% isoflurane. The right common carotid artery was separated and cannulated (1.4 French Micromanometer, Millar Instruments). Left ventricular end-diastolic pressure (LVEDP), Left ventricular end-systolic pressure (LVESP) and heart rate (HR) were measured by advancement of the catheter into the left ventricular cavity. The data were recorded and analyzed on a PowerLab System (USA). These parameters, as well as maximal values of the instantaneous first derivative of left ventricular pressure (+dP/dtmax, a measure of cardiac contractility) and minimum values of the instantaneous first derivative of left ventricular pressure (-dP/dtmax, a measure of cardiac relaxation), were recorded.

Determination of apoptosis

Cardiomyocyte apoptosis in heart tissues was evaluated by using terminal

deoxynucleotidyl transferase dUTP nick-end labeling (TUNEL) staining with an In Situ Cell Death Detection Kit (Roche, 11684817910) according to the manufacturer's instructions.

ADSC apoptosis was evaluated by using a One Step TUNEL Apoptosis Assay Kit (Beyotime, C1090) according to the manufacturer's instructions. Images were acquired with a Nikon Eclipse C1 Microscope and Nikon DS-U3 camera. The apoptosis index was calculated as follows: number of TUNEL-positive nuclei/total number of nuclei. A representative image of each group was selected based upon the mean value.

Analysis of angiogenesis

Heart tissues were dewaxed in water, washed with distilled water, and placed in a water bath with citric acid/sodium citrate buffer solution at room temperature. The samples were incubated in a water bath for 15 minutes after the water bath temperature reached 95°C. After cooling, the slides were permeabilized with 0.3% Triton-100 for 15 minutes, blocked with 1% BSA in PBS for 30 minutes, and incubated with an anti-CD31 primary antibody at 4°C overnight (1 : 100, Cat No. ab28364, Abcam). The secondary antibody was donkey anti-rabbit conjugated with Alexa Fluor 594 (catalog no. 34212ES60, Yeasen, China). The nuclei were stained with 4',6-98 diamidino-2-phenylindole (DAPI, GB1012, Servicebio). Images of immunostained sections were acquired with a Nikon Eclipse C1 Microscope and Nikon DS-U3 camera. The capillary density was quantified by ImageJ software. Only microvessels with a clear lumen structure or linear blood vessel shape were counted. Branching vessel structures were not counted more than once.

Masson's trichrome staining

Hearts were harvested from anesthetized mice and embedded in paraffin, and then, heart tissues were cut into 5-μm-thick sections. The heart tissues were dewaxed in water, washed with distilled water, and placed in a water bath with Bouin's fixative solution. The samples were incubated in the water bath for 15 minutes after the water bath temperature reached 56°C. Then, a Masson's Trichrome Stain Kit (Solarbio, G1340) was used to assess myocardial fibrosis according to the manufacturer's instructions. Photographs were captured with a microscope with a 1.25 objective lens (Nikon, Japan). For quantification, cardiac cross-sectional measurements were analyzed. The average ratio between the fibrosis region and left ventricular region (fibrosis size %) was analyzed by ImageJ software to determine the degree of fibrosis.

Collection of conditioned medium (CM)

When ADSCs reached 70-80% confluence, they were infected with CSF2RB (ADSC-CSF2RB) and NC (ADSC-NC) adenoviruses for 2 days. After 2 days, the cells were washed with PBS and then incubated with α-MEM without FBS and other supplements. Twenty-four hours later, the serum-free α-MEM was collected and centrifuged at 1000 × g for 5 minutes to obtain conditioned medium (CM).

Isolation and quantification of ADSC-derived extracellular vesicles (EVs)

EVs were extracted from the CM of ADSC-NC and ADSC-CSF2RB by using a Total Exosome Isolation Kit for Cell Culture Media (Invitrogen, 4478359) as we have previously described [5]. Briefly, the obtained CM was centrifuged at 3000 × g for 30 minutes and transferred to sterile tubes. Following the addition of the reagent to the CM

and incubation at 4 °C overnight, the mixture was centrifuged at 4 °C and 10000 × g for 1 hour. After the supernatant of the mixture was discarded, the EVs attached to the tube were suspended in PBS.

The EV concentration was assessed with a NanoFCM system. By measuring the number of fluorescent exosomes with a calibrated concentration, the volume flow of the sample under a specific pressure was obtained, and then the standard curve of exosome concentration was generated. Under the same sampling conditions, the exosome concentration of the sample to be tested can be obtained according to the standard curve.

Cell growth assay

ADSCs at passage 2-3 were seeded in 96-well plates with 300 μL of complete medium. Cell viability was measured using a Cell Counting Kit-8 (CCK-8) (Sigma, 96992) per the manufacturer's protocol. The absorbance at 450 nm was read using a SpectraMax M5 microplate reader (Molecular Devices).

Neonatal rat ventricular cardiomyocyte (NRVM) isolation

NRVMs were isolated from 1- to 2-day-old Sprague–Dawley pups following a previously described method with slight modification [6]. Immediately after the euthanasia of the rat pups, the hearts were removed, the ventricles were minced, and the myocytes were isolated with 1.0 mg/mL collagenase type II (Thermo Fisher Scientific, 17101015). The isolated myocytes were collected at 10-minute intervals until the tissues were completely digested. Then, the cells were resuspended in high-glucose DMEM (Sigma, D5796) supplemented with 10% FBS, 10 mM HEPES, and

0.1 mM 5-bromo-2'-deoxyuridine (BrdU, Sigma, B5002), plated in culture dishes, and incubated for 90 minutes to allow the attachment of fast-adherent fibroblasts. Nonadherent cells (ventricular myocytes) were collected, plated in dishes, and cultured in growth media for 48 hours. On the following day, the medium was replaced with M199 supplemented with 0.5% FBS, 10 mM HEPES, and no BrdU.

Determination of capillary-like tube formation

Rat coronary artery endothelial cells (RCAECs) were used to evaluate the tube formation capacity of endothelial cells. Briefly, Matrigel was diluted in serum-free DMEM/F12 medium and then seeded in a 48-well plate. Then, the plate was placed in the incubator to allow the Matrigel to polymerize for 40 minutes. Then, RCAECs at a density of $1 \times 10^4/cm^2$ were seeded onto the Matrigel after resuspension in DMEM without FBS or ADSC-derived conditioned medium and incubated for 4-6 hours. Images of tube formation were obtained with an optical microscope (Nikon, Japan). The total length per field was calculated from five random fields.

Quantitative PCR

Total RNA was extracted from cells or tissues via an RNeasy Mini Kit (Qiagen, 74106). RNA quality and concentration were measured by using a SpectraMax QuickDrop Micro-Volume Spectrophotometer. cDNA was generated from RNA by using the MiniBEST Universal RNA Extraction Kit (#9767, Takara) and a PrimeScript™ RT Reagent Kit with gDNA Eraser (DRR047A, Takara). Then, cDNA was generated from 1 μg of total RNA using the SuperScript III First-Strand Synthesis System (Thermo Fisher Scientific, 18080051) per the manufacturer's protocol. The expression of each

gene was analyzed in duplicate in 10 μl reactions with a PCR detection kit (DRR081A, TaKaRa) and CFX96 system (Bio-Rad). β-actin served as the housekeeping gene. The data were normalized via the standard comparative cycle threshold (CT) method. The primers used in this study were designed and provided by TSINGKE Biotech. All primer sequences are shown in Supplementary Table II.

The thermal cycling conditions were as follows: denaturation at 95°C for 5 minutes followed by 40 cycles of 10 seconds at 95°C, 20 seconds at 55°C, and 20 seconds at 72°C.

Protein extraction

To extract proteins from tissues, myocardial tissues were washed with PBS and centrifuged at 1000 r/min at 4°C for 5 minutes to remove the blood. RIPA protein lysis buffer was added. A tissue homogenizer was used to extract the protein components from the tissues. To extract proteins from cells, an appropriate amount of RIPA protein lysis buffer was added according to the cell density, and the cells were lysed on ice and scraped into an EP tube. An ultrasonic homogenizer was used to extract the protein components from the cells. The concentration of the extracted protein components was quantified by the Bradford method with bovine serum albumin (BSA) as the standard.

Western blot analysis

The proteins were separated on SDS–PAGE gels (10% for RNF4, p-STAT5, STAT5, p-ERK1/2, ERK1/2, p-AKT, AKT, p-JAK2, JAK2 and CSF2RB; 12% for Caspase-3, cleaved caspase-3, CSF2, MMP-2, MMP-3, and MMP-9). Then, the proteins were transferred to a polyvinylidene fluoride membrane. After blocking in 5% milk for 2

hours at room temperature, the membranes were washed three times with 1% TBST buffer and incubated with the corresponding primary antibodies. After incubation with the primary antibodies overnight at 4°C, the membranes were washed three times with 1% TBST buffer and incubated with a secondary HRP-conjugated anti-rabbit antibody (BioCytoSci SA-10011, 1 : 5000) or anti-mouse antibody (BioCytoSci 223 #SA-10010, 1 : 5000) for 1 hour at room temperature. The bands were detected with an enhanced chemiluminescence kit (Millipore, WBKLS0100), and the band densities were quantified with Quantity One software (Bio-Rad). The primary antibodies used in this study were as follows: anti-cleaved caspase-3 rabbit polyclonal antibody (CST #9664) (1/1,000), anti-caspase-3 rabbit polyclonal antibody (CST #9662) (1/1,000), anti-MMP-2 rabbit monoclonal antibody (abcam, #ab52915), anti-MMP-3 rabbit monoclonal antibody (affinity, AF5330) (1/1000), anti-MMP-9 rabbit polyclonal antibody (ab38898) (1/1,000), anti-phospho-Akt rabbit polyclonal antibody (CST #9271) (1/1,000), anti-Akt rabbit monoclonal antibody (CST #4691) (1/1,000), anti-phospho-ERK1/2 mouse monoclonal antibody (CST #9106) (1/1,000), anti-ERK1/2 mouse monoclonal antibody (CST #9107) (1/1,000), anti-CSF2 rabbit monoclonal antibody (affinity #DF12537), anti-RNF4 rabbit monoclonal antibody (Proteintech #17810-1-AP), anti-STAT5 rabbit monoclonal antibody (CST #94205), anti-p-STAT5 rabbit monoclonal antibody (CST #4322), anti-CSF2RB mouse monoclonal antibody (Santa #D2418), and anti-β-actin mouse monoclonal antibody (sc-47778) (1/1,000).

Immunohistochemistry

For the *in vitro* experiment, cells were fixed with 4% paraformaldehyde and

permeabilized in PBS supplemented with 0.2% Triton (Sigma, X-100) for 10 minutes. Then, the cells were blocked with 1% BSA in PBS for 1 hour and incubated overnight with primary antibodies at 4°C.

For fixed tissues, slides were deparaffinized and subjected to antigen retrieval in hot citric acid buffer. After cooling, the slides were permeabilized with 0.2% Triton-100 for 15 minutes, blocked with 1% BSA in PBS for 30 minutes, and incubated overnight with primary antibody at 4°C.

The primary antibodies were probed with donkey anti-rabbit IgG (H+L) secondary antibody conjugated with Alexa Fluor 594 (Cat No. 34212ES60, Yeasen, China), donkey anti-mouse IgG (H+L) secondary antibody conjugated with Alexa Fluor 488 (Cat No. 34106ES60, Yeasen, China), goat anti-mouse IgG (H+L) secondary antibody conjugated with Alexa Fluor 594 (Cat No. 33212ES60, Yeasen, China), and goat anti-rabbit IgG secondary antibody conjugated with DyLight 488 (A23220, ABBKINE). The nuclei in both cells and embedded tissues were stained with 4',6-diamidino-2-phenylindole (DAPI, Vector Laboratories, H-1200). Micrographs of all immunostains were acquired via a Nikon Eclipse C1 Microscope and Nikon DS-U3 camera. A representative image of each group was selected based upon the mean value.

The primary antibodies used in this study included anti-Troponin T mouse monoclonal antibody (Thermo Fisher Scientific, MS-295-P0), anti-CSF2 rabbit polyclonal antibody (Solarbio #K009613P) (1/1,000), anti-RNF4 rabbit monoclonal antibody (Proteintech #17810-1-AP), and anti-p-STAT5 rabbit monoclonal antibody (CST #4322).

References

1. Li Y, Chen B, Yang X, Zhang C, Jiao Y, Li P, et al. S100a8/a9 Signaling Causes Mitochondrial Dysfunction and Cardiomyocyte Death in Response to Ischemic/Reperfusion Injury. Circulation. 2019; 140: 751-64.
2. He Y, Wang R, Liu J, Li F, Li J, Li C, et al. A Randomized Comparison of the Healing Response Between the Firehawk Stent and the Xience Stent in Patients With ST-Segment Elevation Myocardial Infarction at 6 Months of Follow-Up (TARGET STEMI OCT China Trial): An Optical Coherence Tomography Study. Frontiers in cardiovascular medicine. 2022; 9: 895167.
3. Neumann FJ, Sousa-Uva M, Ahlsson A, Alfonso F, Banning AP, Benedetto U, et al. 2018 ESC/EACTS Guidelines on myocardial revascularization. Eur Heart J. 2019; 40: 87-165.
4. Yan W, Guo Y, Tao L, Lau WB, Gan L, Yan Z, et al. C1q/Tumor Necrosis Factor-Related Protein-9 Regulates the Fate of Implanted Mesenchymal Stem Cells and Mobilizes Their Protective Effects Against Ischemic Heart Injury via Multiple Novel Signaling Pathways. Circulation. 2017; 136: 2162-77.
5. Xia Y, Xu X, Guo Y, Lin C, Xu X, Zhang F, et al. Mesenchymal Stromal Cells Overexpressing Farnesoid X Receptor Exert Cardioprotective Effects Against Acute Ischemic Heart Injury by Binding Endogenous Bile Acids. Advanced science (Weinheim, Baden-Wurttemberg, Germany). 2022; 9: e2200431.
6. Yan W, Chen Y, Guo Y, Xia Y, Li C, Du Y, et al. Irisin Promotes Cardiac Homing of Intravenously Delivered MSCs and Protects against Ischemic Heart Injury. Advanced science (Weinheim, Baden-Wurttemberg, Germany). 2022; 9: e2103697.

Online Tables

Table S1: Genes that were upregulated or downregulated in ADSC-CSF2RB compared to ADSC-NC (basal read count > 50, fold change > 1.5 or < 0.67, p < 0.05) identified by RNA sequencing (RNAseq) analysis (14 total)

Gene Name	ADSC-CSF2RB/ADSC-NC	ADSC-CSF2RB/ADSC-CON
CSF2RB	96.48571	120.6071
Ddx41	4.701492537	6.057692308
Lrrk1	4.235294118	26.18181818
Fam118a	2.902298851	1.666666667
C1qa	2.432	5.24137931
Pdzd2	2.152317881	2.519379845
Fah	2.055363322	2.39516129
Cdca4	1.962616822	1.590909091
Avl9	1.950310559	2.136054422
Fam172a	1.68989547	1.632996633
RNF4	1.676691729	1.570422535
Ctrc	1.619387755	1.691897655
Capg	1.539689995	1.776693767
Ccdc148	0.591525424	0.629963899

ADSC-CON, blank control ADSCs; ADSC-NC, ADSCs transfected with adenovirus-control (MOI = 50) for 2 days; ADSC-CSF2RB, ADSCs transfected with adenovirus-CSF2RB (MOI = 50) for 2 days.

Table S2: Real-time PCR primers

Genes	Forward primer (5'—3')	Reverse primer (5'—3')
CSF2	CTGCTTTTGTGCCTGCGTAA	TTGTCTTCCGCTGTCCAAGC
SDF-1	GGGACTTGCTTTGCACAGTT	AAAGGACAAACCTGGGGAGC
HGF	CAAGCAATCCAGAGGTACGC	AAGAACTTGTGCCGGTGTGG
CCL2	AGGTGTCCCAAAGAAGCTGT	GACCTTAGGGCAGATGCAGTT
CCL7	CTTTCAGCATCCAAGTGTGGG	GACCCACTTCTGATGGGCTTC
β-actin	AACAGTCCGCCTAGAAGCAC	CGTTGACATCCGTAAAGACC
Ddx41	TTCACGAATACCTCCTGCTCAAA	CGGTGCACATAGTTCTCGATTTC
Lrrk1	CCAGTTCTGGCTTCTCAACATTG	GCGATCCTCTCTACTCGGAATTT
Fam118a	GCGAGTAGACAGTACCACCTTAC	ACCCTCCAGCATCATCAGTATCT
Pdzd2	TGTCATTTCCATCATCGGGCTATA	CGGATTTGCTTGAAGGTGTGAAT
Fah	CAAACCCAAAGCAGGACCCTAAG	TGGCTCATTCCTTCTCCTTTCAA
Cdca	CAGCCCAGAAACCACTAACTTCT	TGGTCACCACATTTCCTCTTCAG
Avl9	GGCTCAGACCAGACACACTTATT	GGCAAACACAGATATCCCTTTGT
Fam172a	GGGAAGCGGGAAAGGAAAGATAA	GGCTATGAAATGGTCCCACACATA
Rnf4	ATTCAGTGGGCATGAGAGATTGA	TTCGCTTCTGGGTTTGTCTAGAA
Ctrc	TCTGCCTGTAACGGAGATTCTG	ACGGCCTCTTCACAGTTGTATTT
Capg	CGTTTGCCTCTGAACTGCTAATT	AACTGCTTGAAGATGGGACTCTC
C1qa	CCACGGAGGCAGGAACATC	GCTCCCCTCTCTCTCCTTTG
ANP	GAAGATCCAGCTGCTTCGGG	CACACCACAAGGGCTTAGGA
BNP	ATCTCAAGCTGCTTTGGGCA	CACTTCAAAGGTGGTCCCAGA
CSF2RB	AGGACATAGAGTTTGAGGTGGCT	CATAGATGCTGTTGGGTAGGAAT

Online Figures and Figure Legends

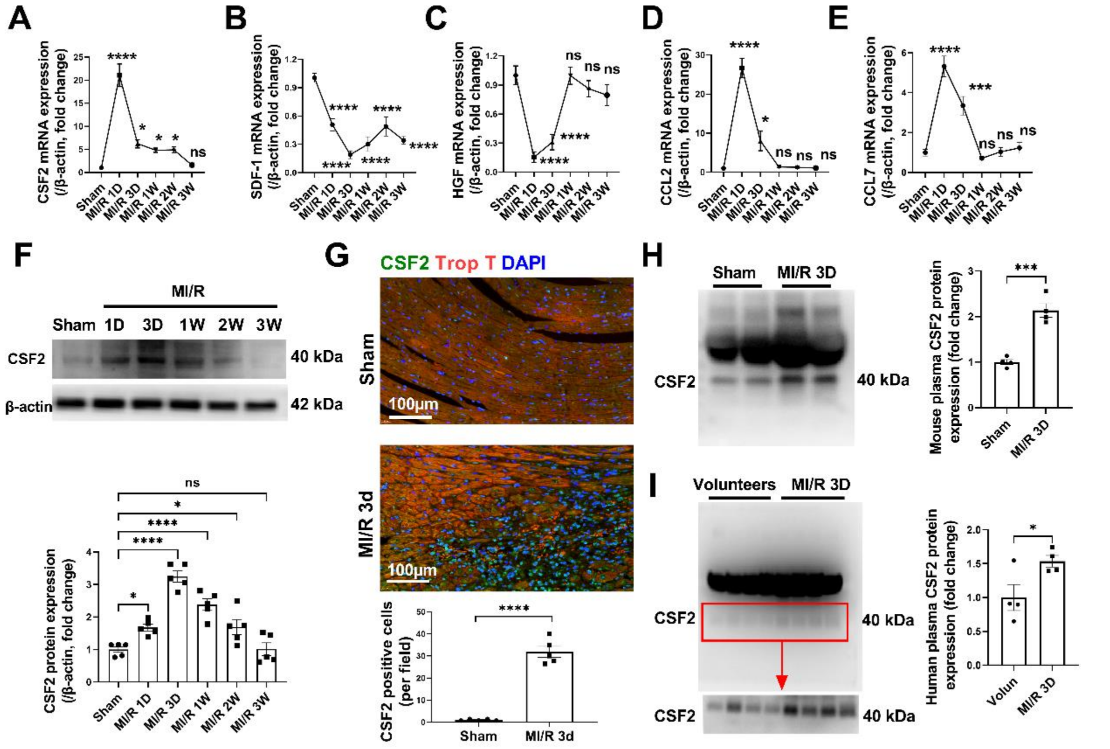

Figure S1. Myocardial CSF2 was upregulated after MI/R. (A-E) mRNA expression of CSF2, SDF-1, HGF, CCL2, and CCL7 in heart tissues at 1, 3, 7, 14, and 21 days after MI/R. n = 5-9 mice per group. (F) Representative immunoblots and protein expression of CSF2 in the peri-infarcted area at 1, 3, 7, 14, and 21 days after MI/R. n = 6. (G) Representative images of CSF2 (green) immunostaining and quantification of the number of CSF2-positive cells in mouse heart sections from the Sham and MI/R groups on day 3. Heart tissues were immunostained for troponin T (green) and DAPI (blue). n = 5 mice. (H) Plasma CSF2 levels in the Sham and MI/R mice were measured by Western blotting after 3 days. n = 4 mice. (I) Human plasma CSF2 levels in non-MI participants and MI/R patients who had acute MI followed by reperfusion therapy were measured after 3 days. n = 4. The data in (A) through (F) were analyzed by 1-way ANOVA followed by Bonferroni post hoc test. The data in (G) through (I) were analyzed by unpaired 2-tailed Student's t test. *p < 0.05, **p < 0.01, ***p < 0.001, ****p < 0.0001. ns, not significant.

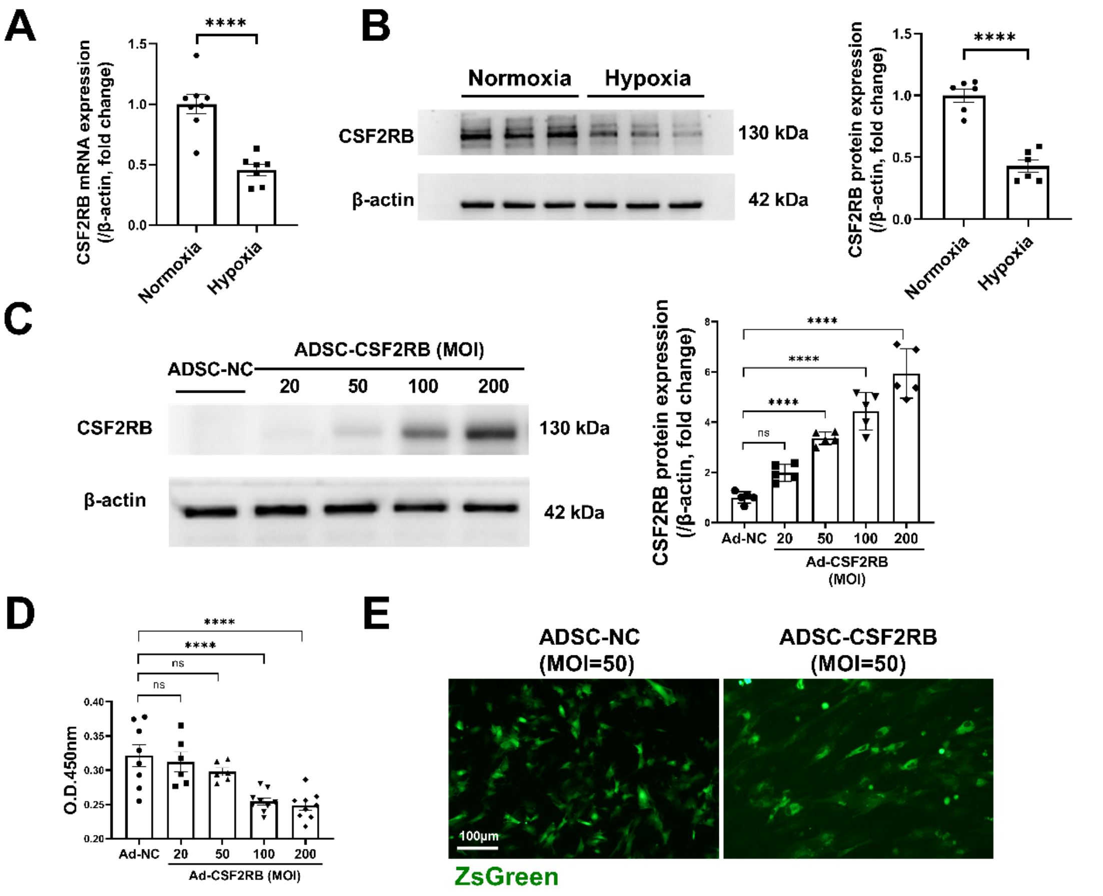

Figure S2. Adenoviruses harboring CSF2RB increased CSF2RB protein expression in ADSCs.

(A) Real-time PCR analysis of the mRNA expression of CSF2RB in ADSCs subjected to normoxia or hypoxia/reoxygenation (H/R). n = 7-8. (B) Western blotting and quantification of the protein expression of CSF2RB in ADSCs. n = 6. The data were analyzed by unpaired, 2-tailed Student's t test. ****p < 0.0001.

(C) Western blotting and quantification of CSF2RB expression in ADSCs 2 days after transfection with control adenovirus (ADSC-NC) or adenovirus carrying CSF2RB (ADSC-CSF2RB) with different multiplicities of infection (MOIs). n = 5. (D) Cell viability of ADSC-NC and ADSC-CSF2RB, as determined by CCK-8 assay. n = 6-8. (E) ZsGreen autofluorescence of ADSC-NC and ADSC-CSF2RB in cells transfected at an MOI = 50. The data were analyzed by 1-way ANOVA followed by Bonferroni post hoc test. *p < 0.05, **p < 0.01, ****p < 0.0001, ns, not significant.

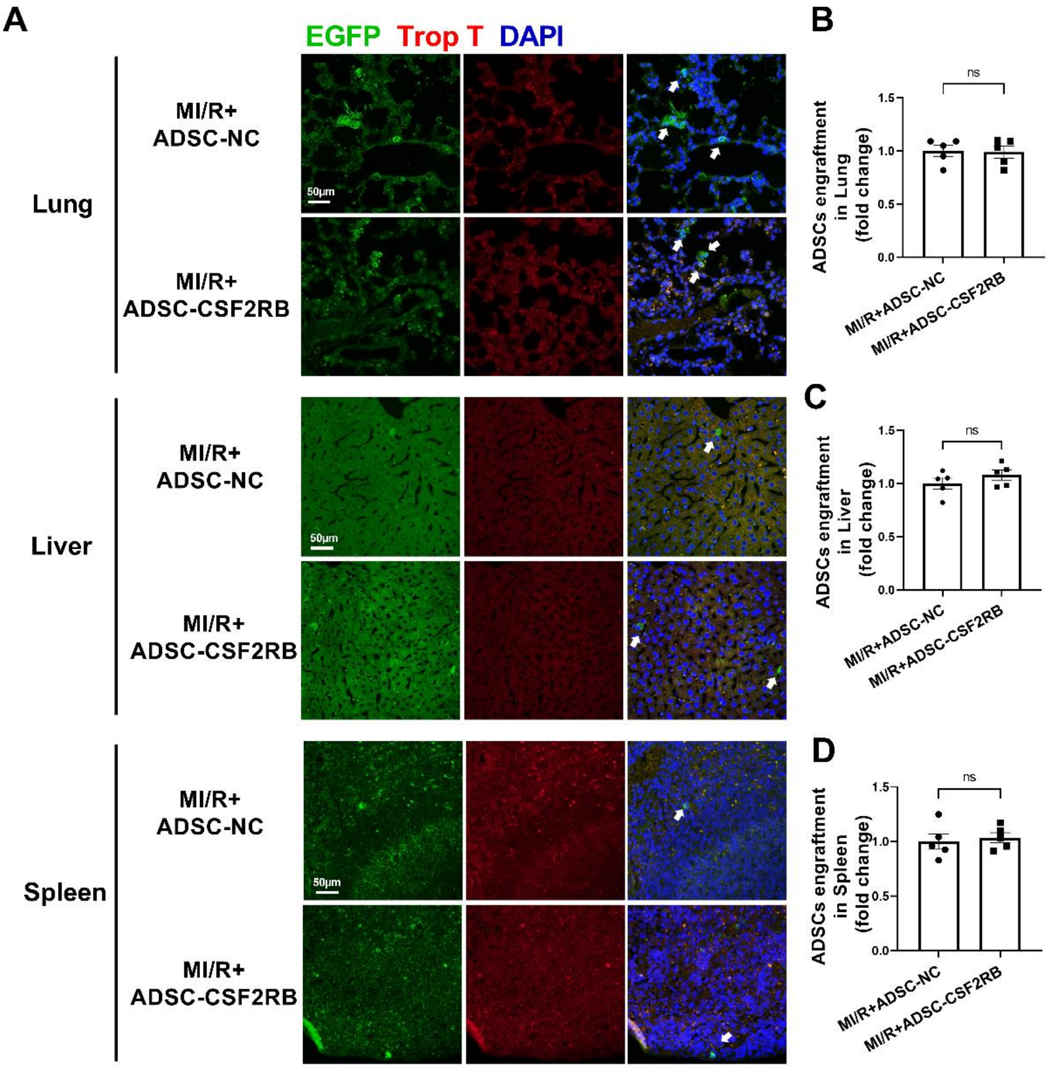

Figure S3. CSF2RB overexpression did not alter ADSC accumulation in the lung, liver, or spleen.

(A-D) Representative images (A) and quantification of the number of EGFP-labeled ADSCs in the lung (B), liver (C), and spleen (D) on day 22 after MI/R. Engrafted ADSCs are positive for GFP expression (green). ADSC-NC, ADSCs transfected with adenovirus-control (MOI = 50) for 2 days; ADSC-CSF2RB, ADSCs transfected with adenovirus-CSF2RB (MOI = 50) for 2 days. n = 5. The data were analyzed by unpaired 2-tailed Student's t test. ns, not significant.

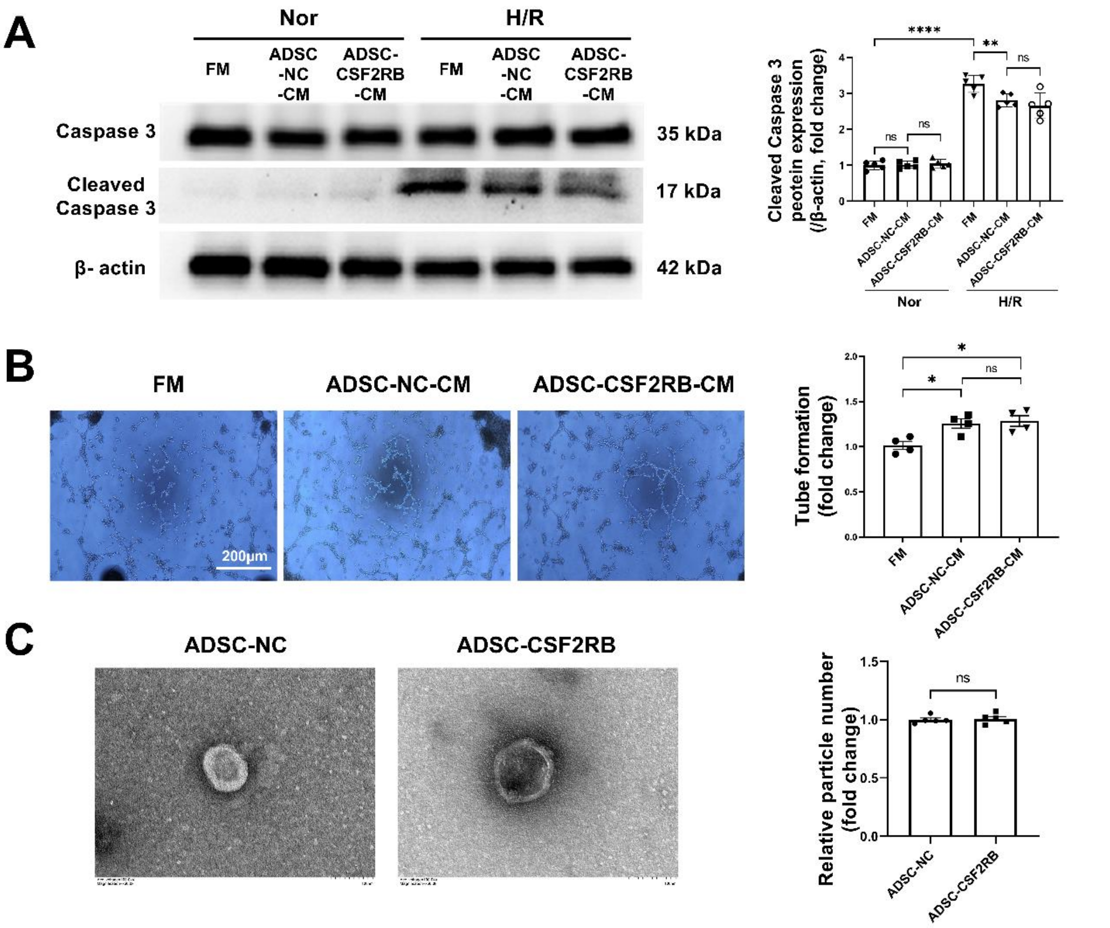

Figure S4. CSF2RB overexpression did not affect the paracrine function of ADSCs.

(A) Protein expression of cleaved caspase-3 in neonatal rat ventricular cardiomyocytes (NRVMs). NRVMs were treated with fresh F12 medium (FM), conditioned medium from ADSC-NC (ADSC-NC-CM), or conditioned medium from ADSC-CSF2RB (ADSC-CSF2RB-CM) 15 minutes before H/R. n = 5.

(B) Tube formation of rat coronary artery endothelial cells (RCAECs) treated with FM, ADSC-NC-CM, or ADSC-CSF2RB-CM for 2.5 hours. n = 4. ADSC-NC, ADSCs transfected with adenovirus-control (MOI = 50) for 2 days; ADSC-CSF2RB, ADSCs transfected with adenovirus-CSF2RB (MOI = 50) for 2 days.

(C) Representative transmission electron microscopy (TEM) images and quantification of ADSC-NC-CM- and ADSC-CSF2RB-CM-derived extracellular vesicles (EVs). n = 5. The data in (A) and (B) were analyzed by one-way ANOVA followed by a Bonferroni post hoc test. The data in (C) were analyzed by unpaired 2-tailed Student's t test. $^{*}P < 0.05$, $^{**}P < 0.01$, $^{****}P < 0.0001$. ns, not significant.

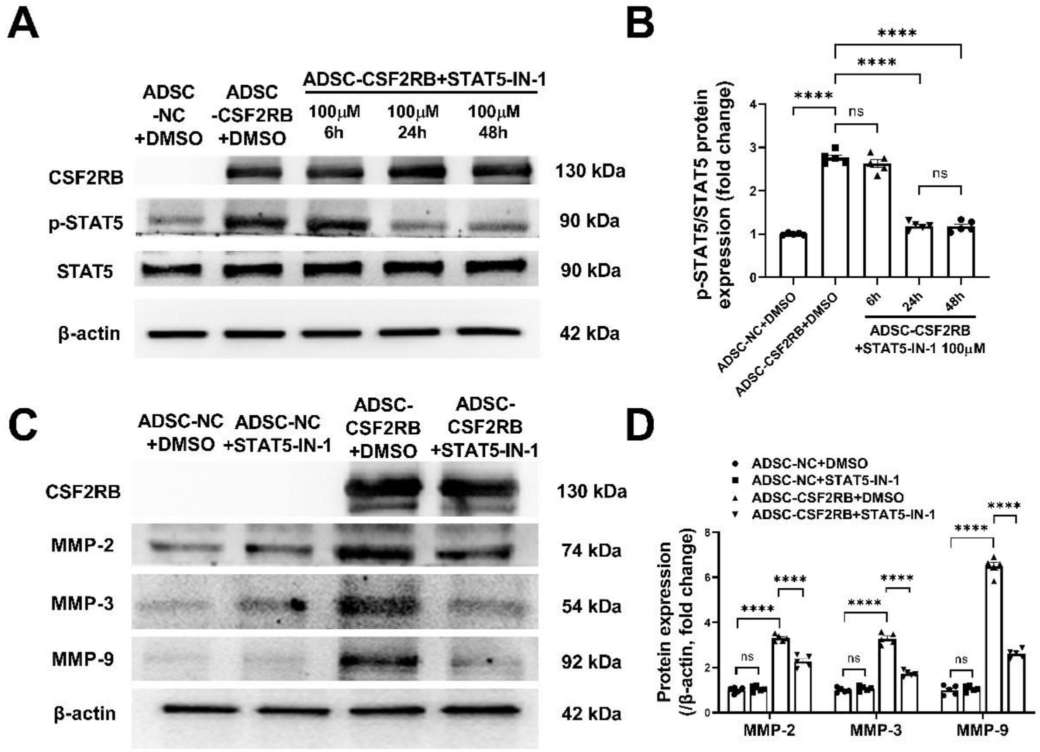

Figure S5. STAT5-IN-1 blocked STAT5 phosphorylation and MMP upregulation in ADSCs overexpressing CSF2RB.

Protein expression (A) and quantification (B) of p-STAT5 in ADSC-NC and ADSC-CSF2RB treated with DMSO or STAT5-IN-1 (100 μM) for 6, 24, and 48 hours. n = 5. (C-D) Protein expression of MMP-2, MMP-3, and MMP-9 in ADSCs. n = 5. ADSC-NC and ADSC-CSF2RB were treated with DMSO or STAT5-IN-1 (100 μM) for 24 hours. ADSC-NC, ADSCs transfected with adenovirus-control (MOI = 50) for 2 days; ADSC-CSF2RB, ADSCs transfected with adenovirus-CSF2RB (MOI = 50) for 2 days. The data were analyzed by one-way ANOVA followed by a Bonferroni post hoc test. ****P < 0.0001. ns, not significant.
